# Synergistic enhancement of the mouse *Pramex1* and *Pramel1* in repressing retinoic acid (RA) signaling during gametogenesis

**DOI:** 10.1186/s13578-024-01212-w

**Published:** 2024-02-23

**Authors:** Mingyao Yang, Francisco Diaz, Ana Rita T. Krause, Yuguo Lei, Wan-Sheng Liu

**Affiliations:** 1 Department of Animal Science, Center for Reproductive Biology and Health (CRBH), College of Agricultural Sciences, The Pennsylvania State University, 311 AVBS Building, University Park, PA 16802 USA; 2 Department of Biomedical Engineering, College of Engineering, The Pennsylvania State University, University Park, PA USA

**Keywords:** *Pramex1*, *Pramel1*, Retinoic acid, Sertoli-cell only, Gene interaction, Gametogenesis, Knockout mice

## Abstract

**Background:**

PRAME constitutes one of the largest multi-copy gene families in Eutherians, encoding cancer-testis antigens (CTAs) with leucine-rich repeats (LRR) domains, highly expressed in cancer cells and gametogenic germ cells. This study aims to elucidate genetic interactions between two members, *Pramex1* and *Pramel1*, in the mouse Prame family during gametogenesis using a gene knockout approach.

**Result:**

Single-gene knockout (sKO) of either *Pramex1* or *Pramel1* resulted in approximately 7% of abnormal seminiferous tubules, characterized by a Sertoli-cell only (SCO) phenotype, impacting sperm count and fecundity significantly. Remarkably, sKO female mice displayed normal reproductive functions. In contrast, *Pramex1/Pramel1* double knockout (dKO) mice exhibited reduced fecundity in both sexes. In dKO females, ovarian primary follicle count decreased by 50% compared to sKO and WT mice, correlating with a 50% fecundity decrease. This suggested compensatory roles during oogenesis in *Pramex1* or *Pramel1* sKO females. Conversely, dKO males showed an 18% frequency of SCO tubules, increased apoptotic germ cells, and decreased undifferentiated spermatogonia compared to sKO and WT testes. Western blot analysis with PRAMEX1- or PRAMEL1-specific antibodies on sKO testes revealed compensatory upregulation of each protein (30–50%) in response to the other gene’s deletion. Double KO males exhibited more severe defects in sperm count and litter size, surpassing *Pramex1* and *Pramel1* sKO accumulative effects, indicating a synergistic enhancement interaction during spermatogenesis. Additional experiments administering *trans*-retinoic acid (RA) and its inhibitor (WIN18,446) in sKO, dKO, and WT mice suggested that PRAMEX1 and PRAMEL1 synergistically repress the RA signaling pathway during spermatogenesis.

**Conclusion:**

Data from sKO and dKO mice unveil a synergistic interaction via the RA signaling pathway between *Pramex1* and *Pramel1* genes during gametogenesis. This discovery sets the stage for investigating interactions among other members within the Prame family, advancing our understanding of multi-copy gene families involved in germ cell formation and function.

## Background

*Preferentially expressed antigen in melanoma* (*PRAME*), also known as PRAME nuclear receptor transcriptional regulator, is a large multi-copy gene family that underwent positive selection and expansion during mammalian evolution [1, 2]. Initially identified as a tumor antigen in human melanoma cells [3], PRAME was subsequently recognized as one of the cancer/testis antigens (CTAs) with roles in both immune response and reproduction [4, 5]. Throughout evolution, the *PRAME* gene family underwent amplification in eutherian mammals, resulting in approximately 60, 60, and 90 copies in the human, cattle, and mouse genomes, respectively [1, 6, 7]. The PRAME family genes encode leucine-rich repeat (LRR) proteins, which adopt a horseshoe-shaped structure that facilitates protein–protein interactions for signal transduction [1, 7]. In the human population, positive selection of *PRAME* alleles may persist, leading to polymorphic copy number variations (CNVs) of the *PRAME* gene among individuals [1]. However, the specific need for multiple copies of *PRAME* and their relationship remains poorly understood.

In rodent and bovid lineages, the *PRAME* gene has transposed to the X and Y chromosomes, respectively, believed to enhance reproductive performance [7, 8]. Notably, CNVs of the *PRAMEY* gene in individual bulls, ranging from 2 to 31 copies, have been significantly associated with testicular size and semen quality [9]. In the mouse genome, the *Prame* gene family is the third largest family, with its members clustered on chromosomes 2, 4, and X [10–16]. The mouse *Prame* gene family exhibits broad expression in the germline at various developmental stages throughout the life cycle [10, 14], suggesting divergence during evolution. Studies on *Pramel7* in embryonic stem cells (ESCs) have highlighted the important role of the Prame family in maintaining naive pluripotency [17, 18]. Additionally, another *Prame* member, *Pramel19* (also known as *Gm12794c*), plays a crucial role in counteracting retinoic acid (RA)-dependent differentiation by repressing the expression of the RA-responsive *Cdkn1A* gene through polycomb repressive complex 2 (PRC2)-mediated transcriptional repression [19]. These findings suggested that the Prame family confers resistance to RA signaling in ESCs, contributing to the maintenance of pluripotency. Postnatally, several *Prame* members are expressed during gametogenesis, exhibiting either testis-specific expression (e.g., *Pramel3*) [14, 15], ovary-specific expression (*Oogenesin1-4*) [20–22], or expression in both male and female gonads (*Pramex2*, *Pramef8 [aka: Pramel12]*, *Pramef12 [aka: Pramel13]*) [10, 14].

Insights into the involvement of the Prame family in spermatogenesis have been obtained through the study of three mouse models: *Pramex1*, *Pramef12*, and *Pramel1* knockout (KO) mice [16, 23, 24]. All these mutant mice display disrupted spermatogenesis, with conditional deletion of *Pramex1* affecting spermatocytes, while globally ablation of *Pramel1* and *Pramef12* exhibit defects in spermatogonia. Abnormal seminiferous tubules with a Sertoli-cell-only (SCO) phenotype was observed in the testes of mutant mice, with a variable degree of severity among the three single gene KO (sKO) mice. Moreover, *Pramex1* and *Pramel1* function in germ cells by inhibiting retinoic acid/retinoic acid receptor (RA/RAR) signaling [24], a function that is similar to their roles in ESCs or cancer cells [19, 24, 25]. While the involvement of various Prame members in gametogenesis was known, the interactions among different members remain uncertain.

During gametogenesis in mammals, RA, a vitamin A derivative, plays multiple roles [26–29]. Mammalian gametes originate from primordial germ cells (PGCs), which colonize the developing gonads and undergo sexual differentiation to generate either oocytes or spermatozoa. Sex determination is triggered by expression of *sex-determining region Y* (*Sry*) in male gonad as early as embryonic day (E) 10.5 in mice, which leads to testis cord development, including differentiation of Sertoli cells [30, 31]. Within the male gonad at E11.5, Sertoli cells synthesize the cytochrome P450 family 26 subfamily B (CYP26B1) [32–34], an enzyme that degrades RA [34, 35]. Germ cells in the testis at E12.5 remain insulated from RA because of CYP26B1 expression and undergo cell cycle arrest without meiosis, whereas germ cells in the ovary respond to RA to initiate meiosis [26, 28, 32, 36]. After birth, male germ cells resume proliferation and undergo a transition from prospermatogonia to spermatogonia, which ultimately develop into haploid spermatozoa through the process of spermatogenesis [37]. The cyclic release of RA by Sertoli cells and germ cells orchestrates three crucial transitions in mouse spermatogenesis: spermatogonial differentiation, meiosis, and spermatid elongation [27, 37]. The importance of RA signaling in both male and female gametogenesis implies the potential involvement of Prame family members in both spermatogenesis and oogenesis, reflecting the intimate connection between the functions of the Prame family and RA signaling.

The objective of this study was to determine whether there is a genetic interaction among the PRAME members under their shared RA signaling pathway during gametogenesis. This study focused on two members: *Prame like, X-linked 1 (Pramex1)* (ID: 75,829), on the X chromosome, and *Prame like 1* (*Pramel1*) (ID:83,491), on chromosome 4 [14, 23]. Previous studies indicated that PRAMEX1 and PRAMEL1 exhibit enriched expression in the germ cells [14, 38]. While the two genes display shared expression patterns in the testes and similar defects in their sKO mouse models, the impact of one mutation in the context of another mutation remains unclear. To ascertain the gene interaction between *Pramex1* and *Pramel1*, we generated and characterized the *Pramex1/Pramel1* double knockout (dKO) mice.

In this study, we found that ablation of either *Pramex1* or *Pramel1* led to compensatory upregulation of the other gene through the shared RA signaling pathway during gametogenesis. The more pronounced defects in reproduction observed in the *Pramex1/Pramel1* dKO mice compared to either *Pramex1* or *Pramel1* sKO mice, suggested a synergistic interaction between *Pramex1* and *Pramel1* during gametogenesis.

## Methods

### Animals

Ethical considerations were strictly followed throughout the study, and all animal procedures were conducted in compliance with the guidelines and regulations set forth by the Animal Care and Use Committees of Penn State University (protocol #46,391). The animals were housed in a controlled environment with a 12-h light–dark cycle, providing them with a suitable diurnal rhythm. They had ad libitum access to food and water, allowing them to maintain optimal hydration and nutrition levels. The founder and wild-type (WT) mice used in the study had a genetic background of C57BL/6. The WT mice with the same genetic background were procured from the Jackson Laboratory (Bar Harbor, ME).

### Generation of *Pramex1/Pramel1* dKO mice

The breeding strategy to produce *Pramex1/Pramel1* dKO mice was outlined in Table 1. In the first generation, we crossed *Pramel1* sKO males (*Pramex1*^+*/Y*^*; Pramel1*^−/−^) with *Pramex1* sKO females (*Pramex1*^*−/−*^*; Pramel1*^+*/*+^). This approach yielded F1 heterozygous offspring with genotypes (*Pramex1*^*−/*+^*; Pramel1*^−/+^) and (*Pramex1*^*−/Y*^*; Pramel1*^−/+^). Then, we crossed the heterozygous offsprings to produce dKO mice in F2 and F3 generations (Table 1).

**Table 1:**
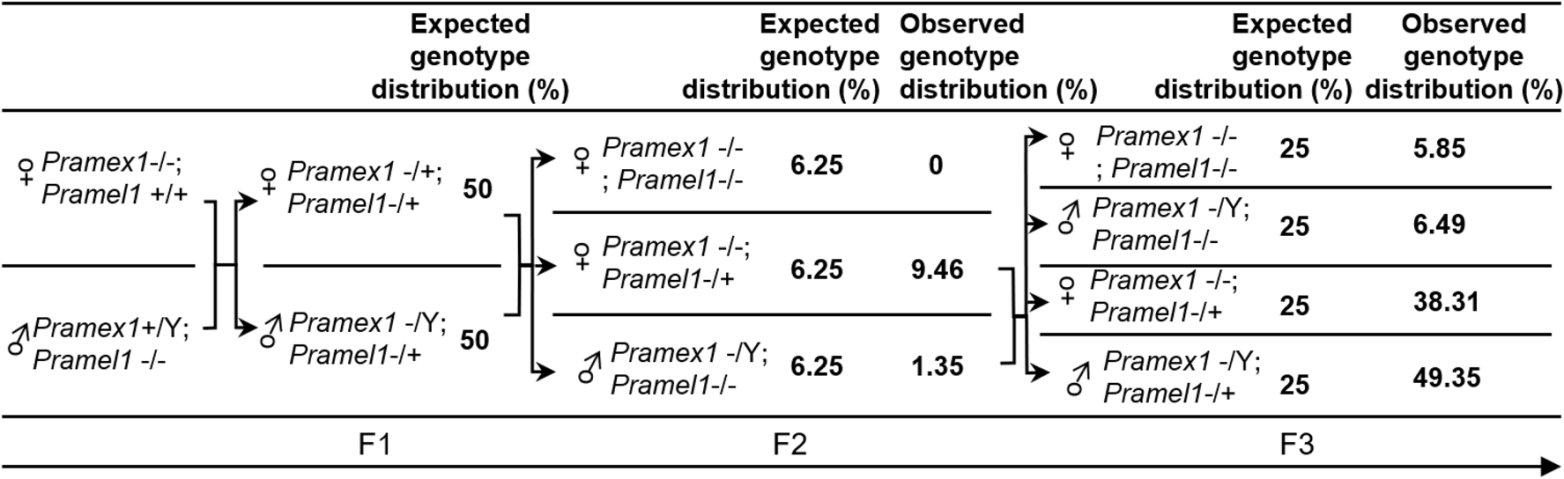
Breeding strategy to produce *Pramex1/Pramel1* dKO mice

### Genotyping.

To determine the genotypes of the mice, genomic DNA was extracted from tail snips using standard procedures. PCR amplification was then performed using specific primer sequences and annealing temperatures, as detailed in Table 2. The PCR reactions were carried out in a total volume of 20 μL, consisting of 10–50 ng genomic DNA, 2.5 μM of each primer, and 0.5 units of Taq polymerase (BIO‐21,105; Bioline, London, UK). The PCR products were subsequently separated by electrophoresis on 1.5% agarose gels containing ethidium bromide in 1X Tris–acetate-EDTA buffer. The gels were visualized and imaged using a GelDocTM XR + Image System, allowing for the detection and documentation of the amplified DNA fragments.

**Table 2:** Primer sequence used for genotyping and RT-PCR

Gene name	Accession number	Primer sequence (5' to 3')	Product length (bp)	Annealing Tm (^o^C)
*Pramel1*	NM_031377	TGGGCTATGTCCATGTATTACCA	185 (WT)/135 (KO)	64
		GTCATCGAGAAGGTCTGCCA		
*Pramex1*	NM_029459	CCAATTCCCCACCTTTTCTT	2270 (WT)/362 (KO)	60
		ACAGCCTGAACCTTGGAGAT		
		CCAATTCCCCACCTTTTCTT	344 (WT)/236 (KO)	60
		TCCTGAAGGCTAAGCCATGT		
*Actb*	NM_007393	GAGAAGCTGTGCTATGTTGCT	72 (WT&KO)	64
		CTCCAGGGAGGAAGAGGATG		

### Testicular weight and sperm analysis

Testes from *Pramex1* sKO, *Pramel1* sKO, *Pramex1/Pramel1* dKO, and WT mice were collected at various time points (P2-P365). The testes were weighed to assess changes in testicular weight over time (n = 3 ~ 5 animals per group). To provide a normalized measurement of testis size, the testis index was calculated as the ratio of the bilateral testicular weight to the body weight, multiplied by 100. For the assessment of sperm counts, the cauda epididymides of P41, P60, P120, and P365 mice were minced in 1 mL of phosphate-buffered saline (PBS). The minced tissue was at 37 °C for 15 min, allowing the spermatozoa to swim out of the epididymides. The resulting suspension was homogenized through gentle pipetting. To facilitate accurate counting, the sperm suspension was diluted with pure water, and the number of spermatozoa was quantified using a hemocytometer. In computer-assisted semen analysis (CASA), the open cauda epididymides were incubated in prewarmed human tubal fluid (HTF) at 37 °C for 5 min, followed by pipetting the sperm suspension onto a CASA slide, and the *SCA® Evolution* CASA program was used to evaluate the sperm motility and concentration [39, 40].

### Mating test

To assess the fecundity of *Pramex1* and *Pramel1* sKO and *Pramex1/Pramel1* dKO male mice, a mating study was conducted. Juvenile male mice, from age of P41, in the experimental groups were paired with mature wild-type (WT) virgin female mice, aged around P60, in co-cages. Similarly, to evaluate the fecundity of female mice, mature WT virgin male mice (at P60) were mated with experimental mature female mice (at P60). Each cage contained one male and one female. The mating rate, indicated by the presence of a copulatory plug, was recorded to determine the success of mating. Additionally, the litter size resulting from each mating was recorded as a measure of reproductive output. During the mating test, we documented the quantity of offspring per litter for every mating and subsequently calculated the mean number of offspring per litter. At least seven mating cages were set up for each genotype to ensure an adequate sample size (n = 6 ~ 7).

### Immunofluorescent staining (IFS) and cell counting

Testes and ovaries from *Pramex1* sKO, *Pramel1* sKO, and *Pramex1/Pramel1* dKO mice at various time points (P7, P14, P21, P35, P120, and P365) with a sample size of n = 3 ~ 5 was fixed overnight in Bouin's solution, followed by embedding in paraffin and sectioning at a thickness of 5 μm. The testis slides were subjected to dewaxing with xylene twice (10 min each) and then sequentially immersed in ethanol baths (100% twice for 10 min, 95%, 70%, and 50% for 5 min each). Antigen retrieval was performed by heating the slides at 95 °C for 15 min in citrate buffer (pH 6.0). After antigen retrieval, the testis sections were blocked in 10% donkey serum in Tris-buffered saline (TBS) supplemented with 0.1% Tween 20 (TBS-T). Subsequently, the sections were incubated overnight at 4 °C in a humid chamber with one or multiple primary antibodies. The primary antibodies used included rabbit anti-SOX9 polyclonal antibody (ab5535; Millipore, Burlington, MA), monoclonal rat anti-TRA98 (#73–003; AS ONE International, Inc. Japan), monoclonal mouse anti-ID4 (B-5, sc-365656; Santa Cruz Biotechnology) or rabbit anti human DDX4 polyclonal antibody (ab13840; Abcam, Cambridge, UK). The primary antibody used for PRAMEX1 (also known as PRAME) detection was obtained from Aviva company (ARP55982_P050). This rabbit polyclonal antibody was raised against the N-terminal region of human PRAME and has a 92% similarity to the mouse homolog. The rabbit PRAMEL1 primary antibody was customized by our laboratory. The primary antibodies were diluted 1:100 in dilution buffer (1% bovine serum albumin and 1% normal donkey serum in TBS-T). Following the primary antibody incubation, the sections were washed three times with TBS-T (10 min each) and then incubated for 1 h at room temperature with the corresponding secondary antibodies. The secondary antibodies used in this study were donkey anti-rabbit immunoglobulin G (IgG) Alexa Fluor 555 (A31572; Thermo Fisher Scientific, Waltham), donkey anti-mouse IgG Alexa Fluor 488 (A21206; Thermo Fisher Scientific), donkey anti-mouse IgG Alexa Fluor 555 (A31570; Thermo Fisher Scientific), donkey anti-rat IgG Alexa Fluor 488 (A21208; Thermo Fisher Scientific), and donkey anti-rat IgG Alexa Fluor 555 (A48270; Thermo Fisher Scientific). The secondary antibodies were diluted at 1:200 in dilution buffer. For dual-staining, two secondary antibodies were mixed in one dilution buffer. Finally, the sections were washed with TBS-T three times for 5 min each, mounted in SlowFadeTM Gold Antifade Mountant with 4',6-diamidino-2-phenylindole (DAPI; S36938; Thermo Fisher Scientific), and analyzed using fluorescence microscopy with an Olympus BX51 microscope. Negative controls (NCs) were obtained by omitting the primary antibody.

To determine the total count of follicles, we tallied the number of cells exhibiting DDX4 staining in the ovarian sections. Cells showing positive DDX4 staining were identified as follicles [41]. Staining and counting were carried out on every fifth section, and the DDX4 + follicle count was multiplied by five to ascertain the overall follicle count per ovary [42, 43].

### Whole-mount seminiferous tubule IFS

Freshly dissected testes were carefully removed from the tunicae, and the seminiferous tubules were gently loosened under dissection microscope with forceps. The testes were then fixed in 4% paraformaldehyde (PFA) overnight at 4 °C. After fixation, the tissues were washed three times with PBS-T (PBS + 1% Triton X-100) by gently rocking for 10 min each at room temperature. Subsequently, the tissues underwent dehydration by incubating in a graded series of ethanol (50%, 70%, 95%, and 100% for 10 min each). Following dehydration, the tissues were rehydrated by incubating in a reverse graded series of ethanol (100%, 95%, 70%, and 50% for 10 min each). Next, the tissues were washed four times in PBS-T for 20 min each. After the washing steps, the tissues were blocked for 1 h in blocking buffer (1% BSA + 0.2% non-fat dry milk powder in PBS supplemented with 0.3% Triton X-100) to reduce non-specific binding. Following blocking, the tissues were incubated overnight at 4 °C in a humid chamber with one or two primary antibodies diluted 1:50 in blocking buffer. The primary antibodies used were rabbit anti-SOX9 polyclonal antibody (ab5535; Millipore, Burlington, MA) or monoclonal rat anti-TRA98 (#73–003; AS ONE International, Inc. Japan). After the overnight incubation, the tissues were washed four times in TBS-T for 20 min each to remove any unbound primary antibodies. Subsequently, the tissues were incubated with the corresponding secondary antibodies for 1 h at room temperature. Following the incubation with secondary antibodies, the tissues were washed in PBS-T three times for 20 min each. Finally, the tissues were mounted with SlowFade™ Gold Antifade Mountant containing 4',6-diamidino-2-phenylindole (DAPI; S36938; Thermo Fisher Scientific) and analyzed using fluorescence microscopy. The imaging was performed using an Olympus BX51 or IX83 microscope. Z-stacks were acquired and flattened using Olympus cellSens (Ver.2.2) imaging software. To quantify the results, the number of germ cells and the length of seminiferous regions were counted and measured based on the flattened images. A sample size of n = 3 ~ 5 was used for analysis. Using the whole-mount IFS images, we measured the length of the SCO region and of the total seminiferous tubules. The SCO frequency was determined by dividing the SCO length by the total length of the seminiferous tubules.

### Oocyte quality measurement and in vitro fertilization (IVF)

Experimental female mice at P25 were intraperitoneally injected with pregnant mare serum gonadotropin (PMSG, 5 IU each; ILEX Life Sciences) to induce follicular development and were euthanized 44 h later by CO_2_ inhalation followed by cervical dislocation, and their reproductive tracts were exposed by opening the abdominal cavity. The ovaries were collected in MEM (Minimum Essential Medium)-alpha supplemented with 2.2 g/L of sodium bicarbonate, 10 µg/mL of streptomycin sulfate, 10 IU penicillin G, 3 mg/mL of bovine serum albumin and 5% fetal bovine serum. The oocytes were released by gentle puncture of ovaries using a 25 G × 5/8 needle to release the cumulus-oocyte complexes. Oocytes surrounded by non-expanded cumulus cells were selected and submitted to 15 h of in vitro maturation (IVM) in collection media supplemented with 10 ng/mL of epidermal growth factor (EGF) at 37 °C in a 5% CO2, 5% O2, 90% N^2^ humidified atmosphere overnight for maturation.

After 15–16 h of oocyte maturation, male mice (at P60-80) were euthanized by CO^2^ inhalation, and cervical dislocation was performed immediately after death. Cauda epididymis was removed and transferred to a one-well dish with 900 μL of homemade MEM medium supplemented with 3 mg/mL of BSA medium (sperm dish). Spermatozoa were released by cutting through the epididymis 2 or 3 times with scissors. The sperm preparation was then incubated at 37 °C in a 5% CO^2^, 5% O^2^, 90% N^2^ humidified atmosphere for 10 min. Sperm count was performed, and the spermatozoa was added to the in vitro fertilization (IVF) dish (4-well NUNC, 500 μL) at a concentration of 2 × 10^6^/mL. The oocytes were quickly washed through homemade MEM wash dishes and transferred to sperm dishes for in vitro fertilization (IVF). It was important to promptly move the expanded cumulus-oocyte complexes with the sperm to avoid zona hardening. After 4 h of co-culture with spermatozoa, the oocytes were washed in homemade MEM & BSA and cultured overnight. At 24 h post fertilization, oocytes were evaluated for fertilization by counting the number of two-cell embryos. Two-cell embryos were cultured in KSOM (Potassium Simplex Optimization Medium) & BSA medium until reaching the blastocyst stage. At 48 h post fertilization, eight-cells embryos could be observed and counted.

### Western blot (WB)

The WB protocol closely followed the prior publication [14]. Briefly, proteins were extracted from testis tissue using CelLytic MT Cell Lysis Reagent (#C3228, Sigma-Aldrich) supplemented with protease and phosphatase inhibitors (#1,860,932 and #1,862,495, Thermo Scientific). The concentration of protein was measured in Nanodrop and a total of 40 µg of protein extracts was used from each sample for WB. The protein from testis were mixed with 4 × Laemmli sample buffer (#1,610,747, BioRad) and 10 × Bolt™ sample reducing agent (#B0009, Life Technologies), followed by denaturation through boiling at 90 °C. These denatured protein extracts were then separated using BioRad Stain Free Gels (#4,568,044, BioRad) and transferred to polyvinylidene difluoride membranes (#IPVH00010, Millipore) using electro-transfer.

After blocking with 5% non-fat dried milk in TBS-T, the membranes were incubated overnight at 4 °C with the primary antibodies: anti-RARα (sc-515796, Santa Cruz Biotechnology), anti-PRAMEX1, anti-PRAMEL1 and anti-YBX2, all at a dilution of 1:500. Following TBS-T washes, the membranes were exposed to anti-rabbit/mouse IgG, HRP-linked secondary antibody (#7074S/#7076S, Cell Signaling Technology) at a 1:1000 dilution for 1 h at room temperature. Reactive proteins were visualized using SuperSignal West Femto Maximum Sensitivity Substrate (#34,095, Thermo Scientific), and WB data were analyzed employing the BioRad ChemiDoc Imaging System. The expected band of proteins for PRAMEX1 (56 kDa), RARα (52 kDa) and YBX2 (48 kDa) were observed.

### Co-immunoprecipitation (co-IP)

For the co-IP experiment, we employed the Pierce Crosslink Magnetic IP/co-IP Kit (#88,805, Thermo Scientific). To elaborate, we immobilized custom-made rabbit anti-PRAMEX1 or monoclonal mouse anti-RARα antibodies onto protein A/G magnetic beads. Subsequently, testis input proteins were introduced to the beads and left to incubate overnight at 4 °C. Following this, the protein complexes were dissociated from the beads for WB analysis using either anti-PRAMEX1 or anti-RARα antibodies, as appropriate.

### RA and WIN18,446 treatment

WT, *Pramex1* sKO, *Pramel1* sKO, and *Pramex1/Pramel1* dKO mice were administered intraperitoneal (i.p.) injections of 10 μL/g body weight of either 0.5 mg/mL all-trans RA (Sigma-Aldrich, Inc.) or 10 μL/g body weight of 2.5 mg/mL WIN18,446 (sc-295819; Santa Cruz Biotechnology) dissolved in 10% DMSO-H2O (n = 3 ~ 4) at postnatal day 2 (P2). At P7, mice testes were collected, and the seminiferous tubules were isolated for further analysis.

### RNA extraction and qRT-PCR

RNA extraction from P7 testis tissue of WT, *Pramex1* sKO, *Pramel1* sKO, and *Pramex1/Pramel1* dKO mice utilized TRlzol reagent (Invitrogen, Carlsbad, CA), and reverse transcription (RT) was executed using the SuperscriptTM III First-Strand Synthesis System (Invitrogen, Carlsbad, CA) following the manufacturer's instructions. The RNA extraction and quantitative real-time PCR (qRT-PCR) procedures were conducted (n = 3) in accordance with a prior study [14]. The sequence-specific primers employed for qRT-PCR amplification of mouse *Pramex1, Pramel1* and *Actb* were provided in Table 2.

### TUNEL assay

Apoptosis was assessed using the In Situ Cell Death Detection Kit, Fluorescein (11,684,795,910; Roche, Penzberg, Germany), following the manufacturer's protocol. The protocol was described in our previous publication [23, 24]. Approximately 300 seminiferous tubules/animal were analyzed, and the number of TUNEL-positive cells was counted for all experimental mice at ages P7, P14, P21, and P35 (n = 3).

### Calculation of referenced anticipated value for the genetic interaction test between *Pramex1 and Pramel1* genes

To test the genetic interaction between *Pramex1* and *Pramel1* dKO genes, we calculated a referenced anticipated value based on the genetic additive effect. This effect considers the independent contributions of different loci to a phenotype, reflecting the combined impact of *Pramex1* and *Pramel1* sKO on spermatogenesis. The referenced anticipated value was determined by multiplying the phenotypic value of *Pramel1* sKO mice by the relative phenotypic value of *Pramex1* sKO mice compared to the WT ($\mathrm{referenced dKO value}=Pramel1 {\text{sKO}}\times \frac{Pramex1\mathrm{ sKO}}{{\text{WT}}}$) [44–46]. We then compared observed and referenced values to assess the severity of phenotypic defects in the dKO. If the deleterious phenotypical value in the dKO was more severe than the referenced anticipated value, we could conclude synergistic genetic enhancement between the two genes, whereas the opposite scenario would indicate genetic suppression [45].

### Statistical analysis

The data were analyzed by the normality test (Shapiro–Wilk test) and equal variance test (Brown–Forsythe) using Sigma Plot 12.0 (Statistical Software). After meeting the assumptions of normally distributed data and homogeneity of variance, the difference in treatment levels was evaluated for significance by Student’s t-test or one-way ANOVA with the post-hoc *Tukey test*. Data were expressed as the mean ± standard error of mean (SEM), and a value of *P* ≤ 0.05 was considered statistically significant. To assess whether the observed phenotypic values align with the expected values, we utilized a Chi-square test for hypothesis testing.

## Results

### Regional SCO seminiferous tubules observed in the *Pramex1* and *Pramel1* sKO testis

**Fig. 1:**
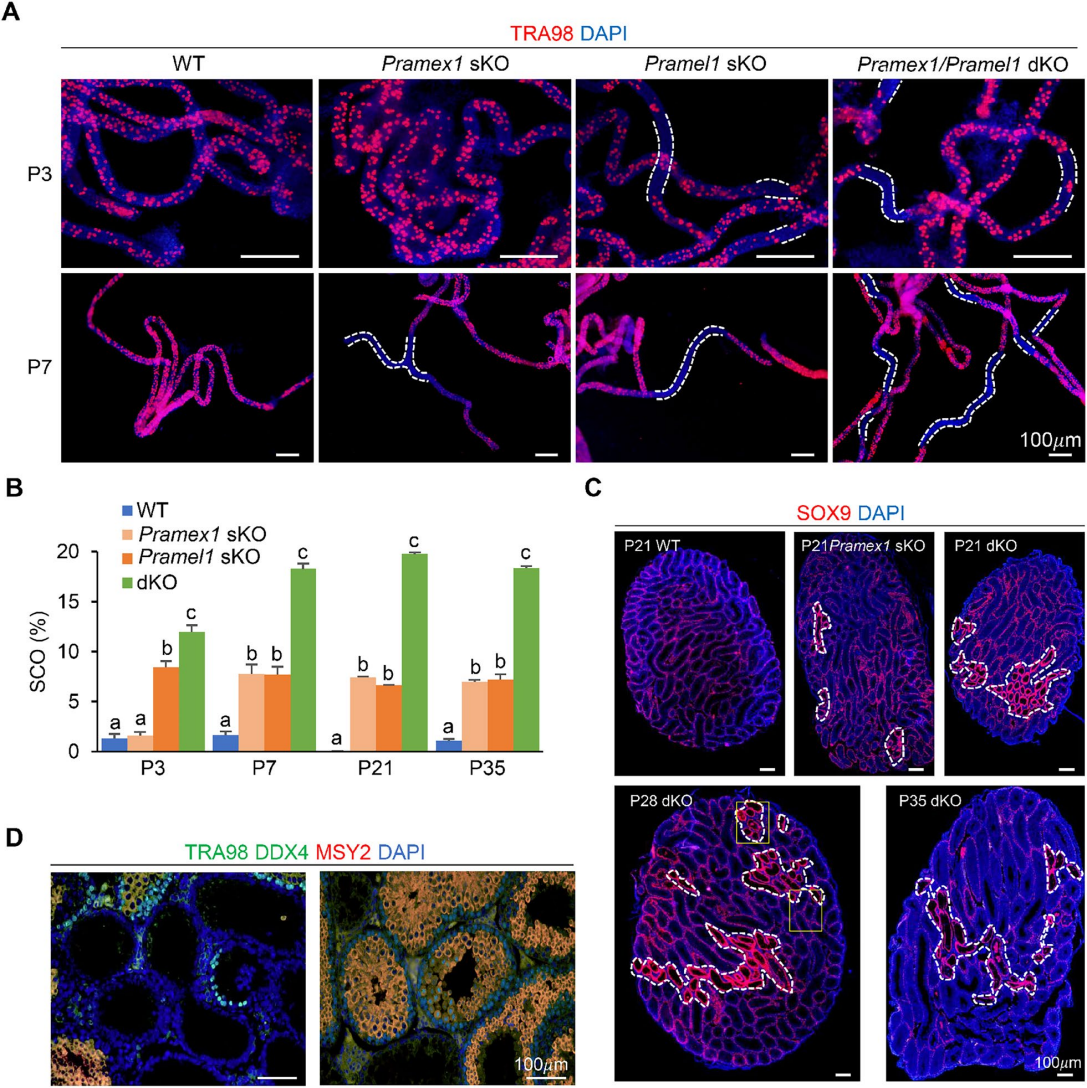
SCO seminiferous tubules observed in the testis of mutant mice. **A** Representative images of whole-mount IFS with TRA98 (red) on seminiferous tubules of WT, *Pramex1* sKO, *Pramel1* sKO, and *Pramex1/Pramel1* dKO mice at P3 and P7. TRA98 was used to label germ cells. The SCO regions lack germ cells and are represented by seminiferous tubules without any TRA98 + cells. White dashed line: SCO regions. Nuclei counterstained with DAPI (blue). Bar = 100 µm.** B** Percentage of SCO segments (%) based on the whole-mount IFS and IFS from P3-35. Significance was assessed among the four groups (WT, *Pramex1* sKO, *Pramel1* sKO, and *Pramex1/Pramel1* dKO mice) for each time point (P3, P7, P21 and P35). Data were expressed as mean ± SEM. Values that do not share a common superscript (a–c) were different significantly (*P* < 0.05). **C** Representative images of IFS with SOX9 (red) counterstained with DAPI (blue) on testis cross-sections of the WT, sKO and dKO (at P21, P28 and P35) mice. SOX9 was a Sertoli cell specific-marker. Dense Sertoli cells were observed along the basement membrane of the SCO tubules where no germ cells were identified (outlined with a white dotted line). The selected regions from the P28 dKO image are enlarged in D. **D** Enlarged images from the P28 dKO section in C. Testis cross-sections from P28 dKO were stained with germ cell-specific markers TRA98 and DDX4, as well as spermatocyte and spermatid-specific marker MSY2. No signal was detected in SCO tubules, whereas strong signals were observed for either TRA98 and DDX4 (green in the left image) or TRA98, DDX4, and MSY2 markers (yellow in the right image) in normal tubules adjacent to SCO tubules. Bar = 100 µm

Abnormal seminiferous tubules, characterized by a SCO phenotype, were observed in both *Pramex1* sKO or *Pramel1* sKO male mice [23, 24]. To determine the frequency of SCO seminiferous tubules, we conducted the whole-mount IFS on seminiferous tubules of neonatal mice with the germ cell-specific marker TRA98. The SCO tubules were identified by the absence of germ cells, as evidenced by the lack of TRA98-positive cells in the whole-mount IFS (Fig. 1A). The occurrence of SCO tubules at P3 and P7 neonatal testis was 1.57 ± 0.08% and 7.78 ± 0.18% in *Pramex1* sKO mice, 7.70 ± 0.52% and 8.42 ± 0.08% in *Pramel1* sKO mice, respectively (Fig. 1A, B). In contrast, WT mice exhibited only ~ 1% (Fig. 1A, B). The rate of SCO tubules was significantly different at P3 (*P* < 0.05) but not at P7 (*P* > 0.05) between *Pramex1* and *Pramel1* sKO mice. These findings suggested that both *Pramex1* and *Pramel1* affect spermatogenesis in neonatal testes albeit potentially impacting germ cells differentially at distinct time points.

SCO tubules in *Pramex1* sKO persisted until P35, in line with previous findings in *Pramel1* sKO testes [24] (Fig. 1B–D). The SCO tubules were clustered rather than randomly distributed across the entire cross-section. By P21 and P35, the frequency of SCO tubules remained consistent in both *Pramex1* sKO (7.42 ± 0.39% and 6.99 ± 0.94%) and *Pramel1* sKO (6.60 ± 0.63% and 7.22 ± 0.81%) mice, respectively (Fig. 1B, C) (*P* < 0.05). The SCO tubules in the testis cross-section were confirmed by SOX9 for Sertoli cells and TRA98, DDX4 and MSY2 for germ cells (Fig. 1C, D). These findings indicated that the single gene ablation of *Pramex1* or *Pramel1* results in a similar occurrence of abnormal seminiferous tubules during spermatogenesis.

### The severe defects in dKO males suggested a synergistic enhancement between the *Pramex1* and *Pramel1* genes

To investigate potential genetic interactions between *Pramex1* and *Pramel1* genes during gametogenesis, we developed a *Pramex1/Pramel1* dKO mouse model (Table 1). We bred the *Pramex1* sKO and *Pramel1* sKO homozygous to produce the heterozygous F1 dKO mice. Theoretically, the F2 progeny from the F1 mating had a 6.25% chance of being dKO females and an equivalent likelihood (6.25%) of being dKO males. However, no dKO females were observed in F2, and only 1.35% of pups had the dKO male genotype (*P* = 4.52 × 10^–8^) (Table 3). To enhance the production of dKO mice, heterozygous females (*Pramex1*^*−/−*^*; Pramel1*^−/+^) were mated with dKO males, which would theoretically yield pups with 25% probability of being either dKO males or dKO females in the F3 generation. Surprisingly, the observed frequency of dKO males and females in F3 generation was only 6.49% and 5.85%, respectively, significantly lower than theoretical prediction (*P* = 1.4 × 10^–14^) (Table 4).

**Table 3:** The number (and percentage) of pups produced in the F2 generation

♀*	♂*									
	*Pramex1*^*−*^*, Pramel1*^*−*^		*Pramel1*^+^*, Y*		*Pramex1*^*−*^*, Pramel1*^+^		*Pramel1*^*−*^*, Y*		Total	
	Actual	Expected	Actual	Expected	Actual	Expected	Actual	Expected	Actual	Expected
*Pramex1*^*−*^*, Pramel1*^−^	0 (0%)	18.5 (6.25%)	18 (6.08%)	18.5 (6.25%)	15 (5.07%)	18.5 (6.25%)	4 (1.35%)	18.5 (6.25%)	37 (12.5%)	74 (25%)
*Pramex1*^+^*, Pramel1*^+^	20 (6.76%)	18.5 (6.25%)	30 (10.14%)	18.5 (6.25%)	27 (9.12%)	18.5 (6.25%)	20 (6.76%)	18.5 (6.25%)	97 (32.77%)	74 (25%)
*Pramex1*^*−*^*, Pramel1*^+^	16 (5.41%)	18.5 (6.25%)	23 (7.77%)	18.5 (6.25%)	25 (8.45%)	18.5 (6.25%)	17 (5.74%)	18.5 (6.25%)	81 (27.36%)	74 (25%)
*Pramex1*^+^*, Pramel1*^*−*^	14 (4.73%)	18.5 (6.25%)	21 (7.09%)	18.5 (6.25%)	28 (9.46%)	18.5 (6.25%)	18 (6.08%)	18.5 (6.25%)	81 (27.36%)	74 (25%)
Total	50 (16.89%)	74 (25%)	92 (31.08%)	74 (25%)	95 (32.09%)	74 (25%)	59 (19.93%)	74 (25%)	296 (100%)	NA

^*^Male and female gametes were produced from a heterozygous female (*Pramex1*^−/+^; *Pramel1*^−/+^) and a heterozygous male (*Pramex1*^−/Y^; *Pramel1*^−/+^)

**Table 4:** The number (and percentage) of pups produced in the F3 generation

♀*	♂*					
	*Pramex1*^*−*^*, Pramel1*^*−*^		*Pramel1*^−^, Y		Total	
	Actual	Expected	Actual	Expected	Actual	Expected
*Pramex1*^*−*^*, Pramel1*^−^	9 (5.85%)	38.5 (25%)	10 (6.49%)	38.5 (25%)	19 (12.34%)	77 (50%)
*Pramex1*^+^*, Pramel1*^*−*^	59 (38.31%)	38.5 (25%)	76 (49.35%)	38.5 (25%)	135 (87.66%)	77 (50%)
Total	68 (44.16%)	77 (50%)	86 (55.84%)	77 (50%)	154 (100%)	

^*^Male and female gametes were produced from a heterozygous female (*Pramex1*^−/+^; *Pramel1*^−/−^) and homozygous male (*Pramex1*^−/Y^; *Pramel1*^−/−^)

**Fig. 2:**
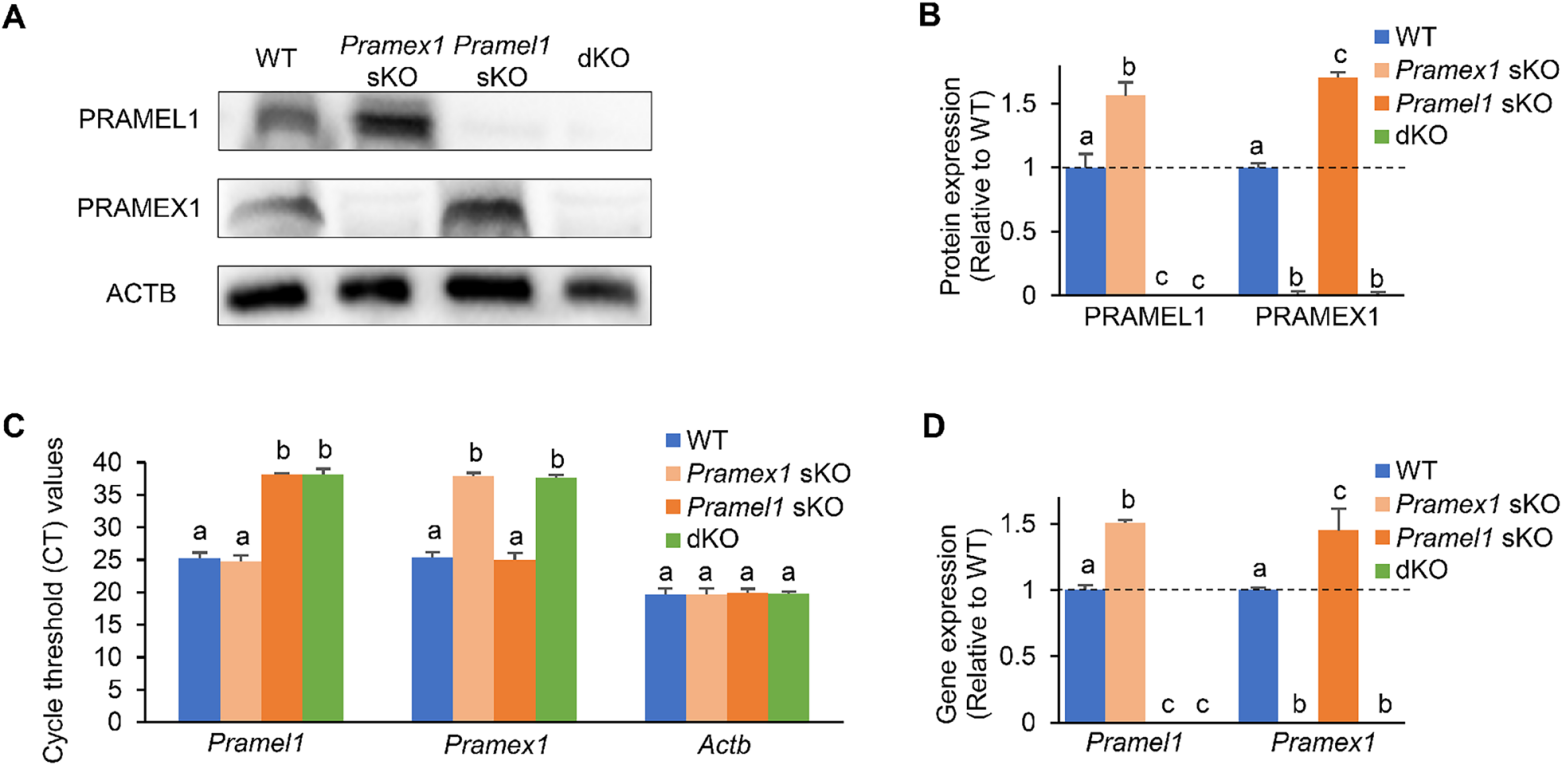
Deletion of PRAMEL1 and PRAMEX1 in the *Pramex1/Pramel1* dKO mice. **A** Western blot (WB) was used to detect the PRAMEL1 and PRAMEX1 expression in the WT, *Pramex1* sKO, *Pramel1* sKO, and *Pramex1/Pramel1* dKO mice at P7. **B** Relative expressions of PRAMEL1 and PRAMEX1 in the *Pramex1* sKO, *Pramel1* sKO, and *Pramex1/Pramel1* dKO mice to WT (WT was set as 1) were detected in WB. **C** Cycle threshold (CT) value of qRT-PCR for *Pramel1*, *Pramex1* and *Actb* genes was compared between the WT, *Pramex1* sKO, *Pramel1* sKO, and *Pramex1/Pramel1* dKO mice. CT values above 37 indicate minimal amounts or none of target nucleic acid. **D** Relative expression of *Pramel1* and *Pramex1* in the *Pramex1* sKO, *Pramel1* sKO, and *Pramex1/Pramel1* dKO mice to WT (WT was set as 1) were detected by qRT-PCR. PRAMEL1 was not deleted in *Pramel1* sKO and *Pramex1/Pramel1* dKO mice and ablation of PRAMEX1 in *Pramex1* sKO and *Pramex1/Pramel1* dKO mice. In addition, higher expression of PRAMEL1 and PRAMEX1 was observed in *Pramex1* sKO and *Pramel1* sKO, respectively. Significance was assessed among the four groups (WT, *Pramex1* sKO, *Pramel1* sKO, and *Pramex1/Pramel1* dKO mice) for each antibody (anti-PRAMEL1 and anti-PRAMEX1). Data were expressed as mean ± SEM. Values that do not share a common superscript (a–c) were found to differ significantly (*P* < 0.05)

In addition to PCR-genotyping, Western blotting (WB) was applied to exam whether PRAMEX1 or PRAMEL1 protein was expressed in the sKOs and dKO testes at P7. The PRAMEL1- (or PRAMEX1-) specific antibody detected a ~ 57 kDa (or ~ 56 kDa) band (expected molecular weight for the mouse PRAMEL1 or PRAMEX1) in WT and *Pramex1* (or *Pramel1*) sKO mice, but not in *Pramel1* (or *Pramex1*) sKO and *Pramex1/Pramel1* dKO testis (Fig. 2A), confirming the deletion of the protein in the corresponding mutant mice. Interestingly, the PRAMEX1 protein expression was 1.71-fold higher in the *Pramel1* sKO testis when compared to WT (*P* < 0.01) (Fig. 2B). Similarly, the PRAMEL1 protein was 1.56-fold higher in the *Pramex1* sKO testis in comparison to WT (*P* < 0.01) (Fig. 2B). These results suggested that the ablation of either *Pramex1* or *Pramel1* may induce an upregulated expression of the other gene in the testis. The gene deletion in three mutant mice at P7 and the upregulation of the other gene in the two sKO mice were confirmed at the gene transcriptional level by qRT-PCR with *Pramex1- and Pramel1-*specific primers (Fig. 2C, D). This compensatory expression has the potential to alleviate the impact of individual mutations, implying that more pronounced defects could manifest when both genes are simultaneously deleted.

To determine the functional interaction of the *Pramex1* and *Pramel1* genes, we characterized the phenotypes of the dKO mice. As shown in Fig. 1, at P3 and P7, compared to both *Pramex1* (1.57 ± 0.08%; 7.78 ± 0.18%) and *Pramel1* (8.42 ± 0.08%; 7.70 ± 0.51%) sKO mice, the dKO testis showed a higher frequency of SCO tubules (*P* < 0.01), with 11.97 ± 0.18% and 18.29 ± 0.78% respectively. The elevated SCO tubule frequency in dKO mice at P7 compared to P3 coincided with the period of SCO region formation in *Pramex1* sKO mice. In neonatal testis at P3 and P7, the SCO frequency in male dKO mice was 18–20% higher than the combined frequency in *Pramex1* sKO and *Pramel1* sKO males, suggesting synergistic enhanced defects in the dKO mice compared to sKO mice. Consistent with the SCO frequency in neonatal testis, the frequency of SCO tubules in the testes of dKO mice remained consistently high at approximately 18–20% during the first round of spermatogenic process from P21 to P35 (Fig. 1B–D).

**Fig. 3:**
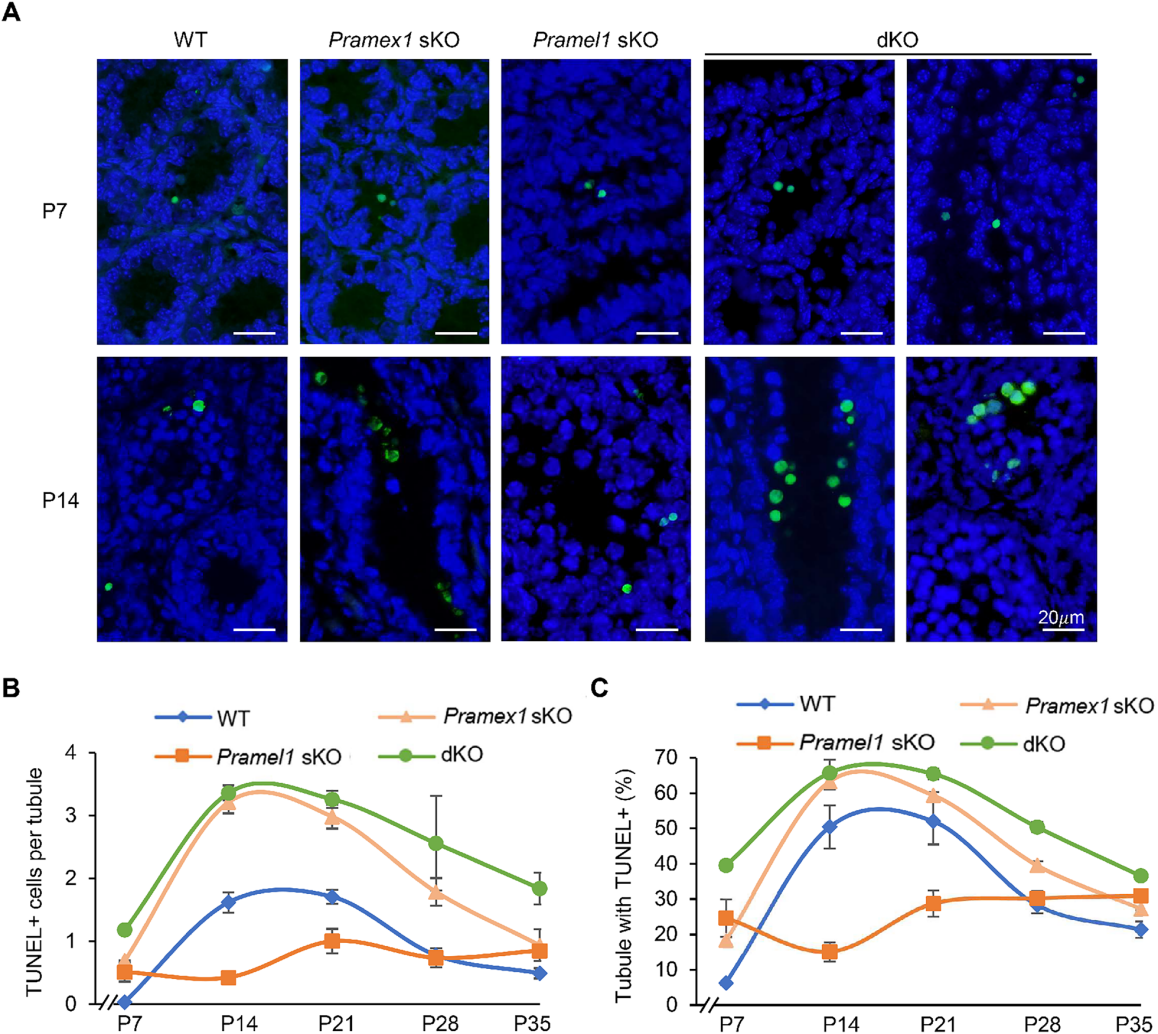
Germ cell apoptosis was increased in the sKO and dKO testis. Testis was evaluated at P7, P14, P21, P28 and P35 and apoptosis was assessed by TUNEL assay in the WT, *Pramex1* sKO, *Pramel1* sKO, and *Pramex1/Pramel1* dKO mice. **A** Representative TUNEL (green) staining of testis sections in the WT, *Pramex1* sKO, *Pramel1* sKO, and *Pramex1/Pramel1* dKO mice at P7 to P15. More apoptotic cells occur in the lumen of seminiferous tubules in the dKO than sKO and WT mice. Bar = 20 µm.** B** Number of TUNEL-positive (TUNEL^+^) cells in the mouse seminiferous tubules for the four groups of mice. **C** Percentage of seminiferous tubules with apoptotic cells (TUNEL^+^). Apoptotic cells were significantly increased in the dKO mice than that sum of *Pramex1* sKO and *Pramel1* sKO. Data were expressed as mean ± SEM

Apart from the SCO tubules, the dKO mice displayed a greater quantity of apoptotic cells compared to the numbers observed in the sKO mice for *Pramex1* and *Pramel1*. At P7, dKO mice showed a higher number of TUNEL + cells per tubule (1.18 ± 0.06) than *Pramex1* sKO (0.69 ± 0.01) and *Pramel1* sKO (0.51 ± 0.15) mice (*P* < 0.01) (Fig. 3A, B). Similarly, the percentage of tubules with TUNEL + cells in the dKO (39.49 ± 1.34) at P7 was close to the sum of *Pramex1* (18.40 ± 1.03) and *Pramel1* sKO (24.66 ± 5.31) mice (Fig. 3C). Like *Pramex1* sKO and WT mice, dKO mice displayed a peak of germ cell apoptosis at P14 (Fig. 3A, B). The apoptotic results, including the number of TUNEL + cells per tubule (3.36 ± 0.12) and the percentage of tubules with TUNEL + cells (65.7 ± 3.70), in the dKO mice at P14 were similar to those in *Pramex1* sKO mice (*P* > 0.05) but higher than those in *Pramel1* sKO and WT mice (*P* < 0.01) (Fig. 3C). A 2.1-fold increase in TUNEL + cells and a 30% higher percentage of tubules with TUNEL + cells were observed in the dKO compared to WT mice at P14. At P21, apoptotic cell numbers were comparable to those at P14 in all four groups (Fig. 3B, C). Similarly, at P14, both dKO and *Pramex1* sKO males exhibited higher apoptotic cell counts compared to WT and *Pramel1* sKO mice at P21. As the mice progressed to P28 and P35, there was a general decrease in apoptotic cell numbers across all groups. In conclusion, the higher incidence of abnormalities, such as SCO tubules and apoptotic cells, observed in the dKO mice was anticipated to lead to more pronounced phenotypic defects.

**Fig. 4:**
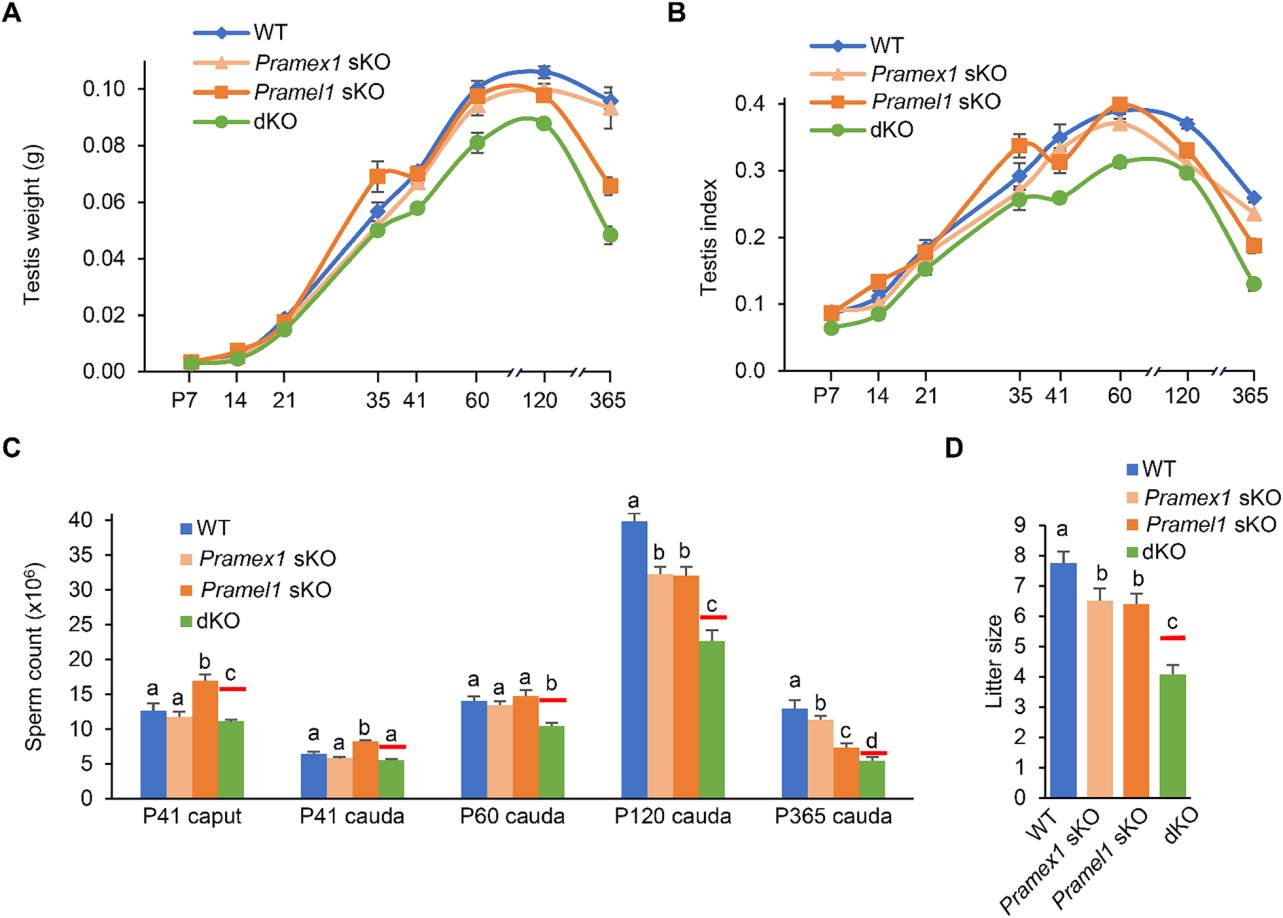
Severe reproductive defects observed in the *Pramex1/Pramel1* dKO mice. **A** Testis weight of WT, *Pramex1* sKO, *Pramel1* sKO, and *Pramex1/Pramel1* dKO mice obtained during the time-course study from P7 to P365. **B** Testis index (= testicular weight (g) / body weight (g) × 100) was documented in WT, *Pramex1* sKO, *Pramel1* sKO, and *Pramex1/Pramel1* dKO mice from P7 to P365 (n = 3 ~ 5). **C** Sperm count in epididymis of WT, *Pramex1* sKO, *Pramel1* sKO, and *Pramex1/Pramel1* dKO mice at P41 (caput and cauda), P60 (cauda), P120 (cauda) and P365 (cauda) of age (n = 3 ~ 5). The referenced sperm count of the dKO was drawn as red solid line in the figure. The formula for the referenced anticipated value of dKO, which calculate effect of two genes deletion without gene interaction in the additive model, was as follows: ($\mathrm{reference dKO value}=Pramel1 {\text{sKO}}\times \frac{Pramex1\mathrm{ sKO}}{{\text{WT}}}$[44–46] (n = 3 ~ 5). **D** Litter size of mating tests of male fecundity. The experimental male mice (P60) were mated with adult WT female. The referenced litter size of the dKO was drawn as red solid line. About 6 ~ 7 male mice were tested and about 30 litters were counted for each group. Significance was assessed among the four groups (WT, *Pramex1* sKO, *Pramel1* sKO, and *Pramex1/Pramel1* dKO mice) for **C** and **D**. Data were expressed as mean ± SEM. Values that do not share a common superscript (a–d) were found to differ significantly (*P* < 0.05)

To further validate the compensatory capacity of the two genes and comprehend the impact of a mutation in one gene on the other, we assessed various phenotypes in the dKO males, including testis weight, testis index, sperm counts, and litter size, and compared the observed value with a referenced value based on a gene additive genetic model (see Methods). The testis weight and testis index of dKO mice were significantly lower than sKOs and WT (Fig. 4A, B) (*P* < 0.05). Furthermore, we assessed caput and cauda sperm production from the first round of spermatogenesis at P41, and from subsequent rounds of spermatogenesis in the cauda at P60, P120, and P365. The dKO males displayed a reduction in sperm count ranging from 12 to 58% compared to WT, which were 13–29% lower than referenced value at all ages studied (*P* < 0.05) (Fig. 4C). The decrease in sperm count in dKO males at P120 was confirmed by CASA analysis, though no significant difference were found in sperm motility between dKO and WT mice (Table 5). Additionally, all sKO, dKO and WT males, from the age of P41, were paired with adult WT females to evaluate their fecundity. The average number of offspring per litter, produced by *Pramex1* sKO, *Pramel1* sKO, dKO and WT males (n = 6 ~ 7), was 6.50 ± 0.41, 6.39 ± 0.36, 4.08 ± 0.30, and 7.76 ± 0.38, respectively (Fig. 4D). The number of offspring produced by dKO males was reduced by 47.41% compared to WT males (*P* < 0.01) (Fig. 4D). These observations were significantly lower than the referenced anticipated value (Fig. 4C-D) suggesting a synergistic enhancement between the two genes.

**Table 5:** Cauda sperm count and motility analysis using CASA at P120

	Rapid progressive (%)	Medium progressive (%)	Non progressive (%)	Motile (%)	Immotile (%)	Sperm count (million/animal)
WT	31.85	28.73	18.23	78.81	21.19	42.75
dKO	42.62	25.11	13.08	80.81	19.19	21.65
*P*-value	0.2285	0.3586	0.1712	0.3914	0.3913	0.0067

### Reduction of undifferentiated spermatogonia in *Pramex1/Pramel1* dKO mice

**Fig. 5:**
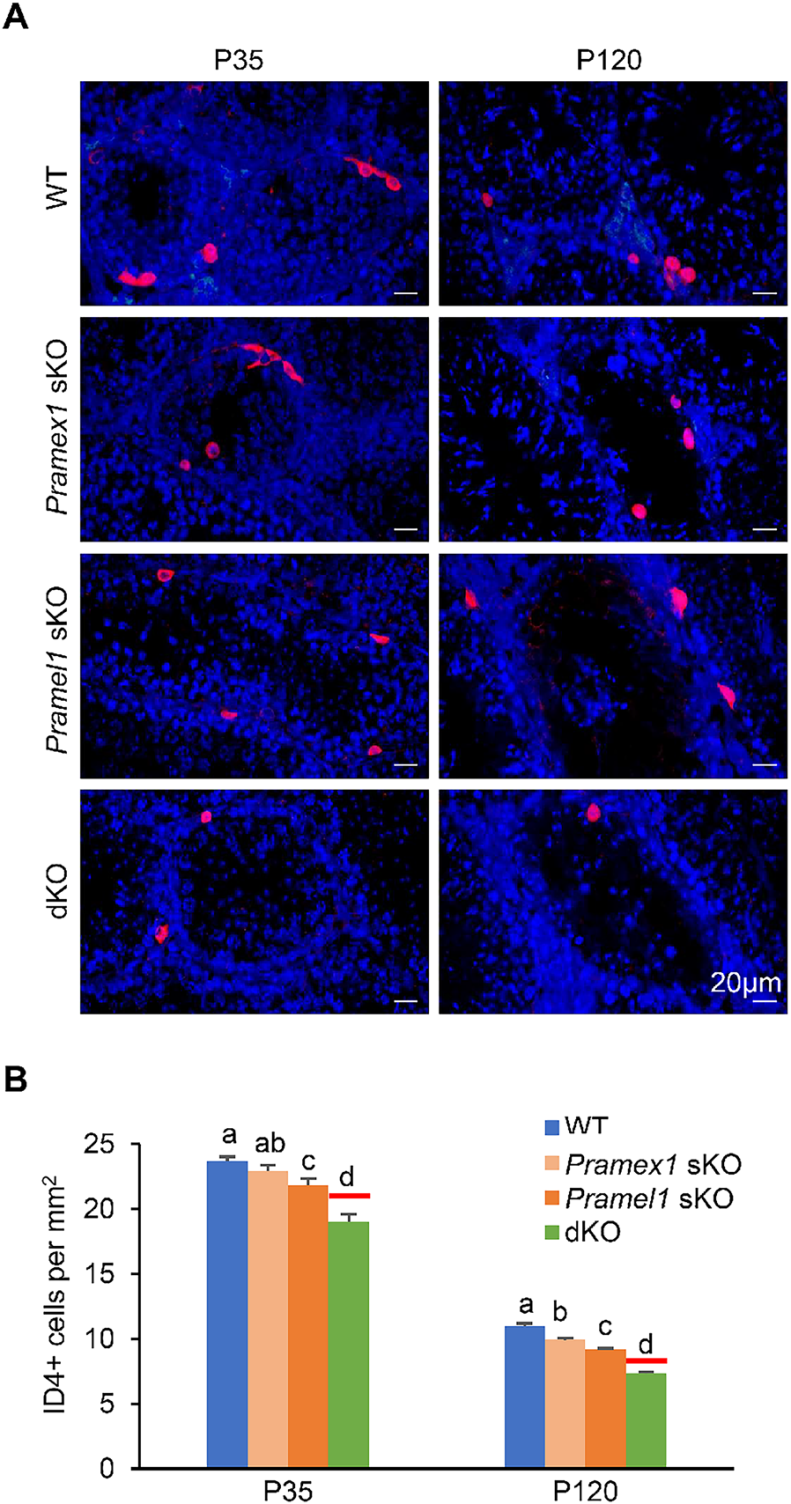
Reduced number of undifferentiated spermatogonia in the *Pramex1/Pramel1* dKO mice.** A** Representative images of IFS with ID4 (red) in testis cross-sections for WT, *Pramex1* sKO, *Pramel1* sKO, and *Pramex1/Pramel1* dKO mice at P35 and P120 (n = 3). Bar = 20 µm. **B** Number of ID4 + spermatogonia/mm^2^ based on IFS result for the four groups at P35 and P120. The referenced number of ID4 + undifferentiated spermatogonia in the dKO was drawn as red solid line. The IFS experiments in A-B were replicated three times. Significance was assessed among the four samples (WT, *Pramex1* sKO, *Pramel1* sKO, and dKO mice) at two ages (P35 or P120). Data were expressed as mean ± SEM. Values that do not share a common superscript (a–d) were found to differ significantly (*P* < 0.05)

To understand the cellular mechanism behind the elevated rate of SCO tubules and reduced sperm count in the dKO mice, we quantified undifferentiated spermatogonia (ID4 + cells/mm^2^) at two developmental stages, juvenile (P35) and mature (P120) (Fig. 5A). At P35, both *Pramel1* sKO (21.86 ± 0.48/mm^2^) and dKO (19.01 ± 0.60/mm^2^) mice had significantly fewer undifferentiated spermatogonia compared to the WT mice (23.71 ± 0.32/mm^2^) (*P* < 0.01) (Fig. 5B), whereas difference between the *Pramex1* sKO (22.91 ± 0.47/mm^2^) and WT mice was not statistically significant (*P* > 0.05). While the *Pramel1* sKO mice exhibited an 8% reduction in undifferentiated spermatogonia compared to the WT mice (*P* < 0.01), the dKO mice displayed a significantly more pronounced decrease (20%) (*P* < 0.01), which was 10% lower than the referenced value (Fig. 5B) (*P* < 0.01). With the progression of aging in the mice, we noted a progressively scattered arrangement of undifferentiated spermatogonia, attributed to the elongation of seminiferous tubules. This phenomenon led to a reduction in the quantity of ID4 + cells within a given unit size at P120 compared to P35 (Fig. 5A, B). At P120, the dKO mice (7.31 ± 0.14/mm^2^) as well as both the *Pramex1* sKO (9.93 ± 0.16/mm^2^) and *Pramel1* sKO (9.19 ± 0.09/mm^2^) mice had significantly fewer ID4 + cells compared to the WT mice (10.98 ± 0.21/mm^2^) (*P* < 0.01) (Fig. 5A, B). The dKO mice exhibited a 33.41% reduction in ID4 + cells than that of the WT mice, while *Pramex1* sKO and *Pramel1* sKO mice showed reductions of 9.61% and 16.35%, respectively (*P* < 0.01) (Fig. 5A, B). The referenced undifferentiated spermatogonia number for the dKO was 8.30 ± 0.08/mm^2^, which was 12% more than what was observed in dKO mice (*P* < 0.01) (Fig. 5B). These results indicated that the simultaneous ablation of both genes leads to a greater reduction in the ability of male mice to maintain the undifferentiated spermatogonia compared to the combined effects of ablating either one of the genes, confirming the synergistic enhancement between the two genes at the cellular level.

### Synergistic repression of RA/RAR signaling by *Pramex1* and *Pramel1* during spermatogenesis

**Fig. 6:**
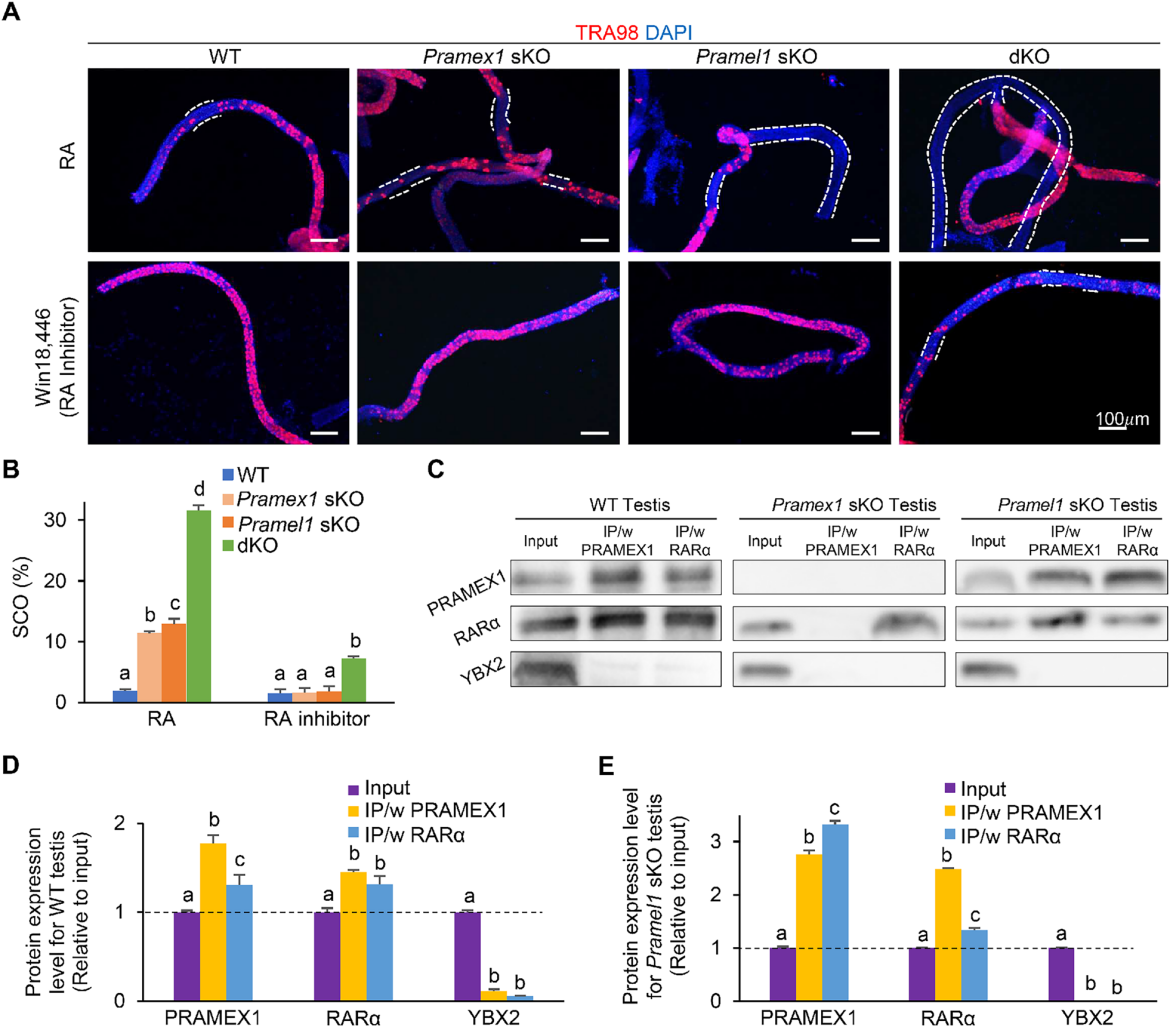
The synergistic enhancement between *Pramex1* and *Pramel1* was through retinoic acid (RA) signaling pathway. **A** Representative images of whole-mount IFS with TRA98 (red) on seminiferous tubules of WT, *Pramex1* sKO, *Pramel1* sKO, and *Pramex1/Pramel1* dKO mice at P7, these animals were treated with RA or WIN18,446 (RA inhibitor) at P2. TRA98 was used to label germ cells. The SCO regions, labeled with white dashed line, lacked germ cells which were seminiferous tubules without any TRA98 + cells. Nuclei counterstained with DAPI (blue). Bar = 100 µm. **B** SCO segments along the length of seminiferous tubules (%) based on the whole-mount IFS for the RA/WIN18,446 treatment in the four groups (n = 3). Significance was assessed among the four groups (WT, *Pramex1* sKO, *Pramel1* sKO, and *Pramex1/Pramel1* dKO mice) for each treatment (RA or RA inhibitor). **C** Co-immunoprecipitation (co-IP) analyses of PRAMEX1 and RARα in P7 WT, *Pramex1* sKO and *Pramel1* sKO testis tissue. YBX2 served as a control. **D** Based on the band intensity in the WB for WT testis tissue, the protein expression level relative to input WT testis (set as 1) was measured (n = 3). Significance was assessed among the three samples (input, IP/w PRAMEX1, and IP/w RARα) for each antibody (PRAMEX1, RARα and YBX2). **E** Based on the band intensity in the WB, the protein expression level relative to input *Pramel1* sKO testis (set as 1) was measured (n = 3). Significance was assessed among the three samples (input, IP/w PRAMEX1, and IP/w RARα) for each antibody (PRAMEX1, RARα and YBX2). Data were expressed as mean ± SEM. Values that do not share a common superscript (a–d) were found to differ significantly (*P* < 0.05)

Our previous studies indicated that both *Pramex1* and *Pramel1* function in spermatogenesis by repressing the RA signaling [23, 24]. To understand how the synergistic interactions of *Pramex1* and *Pramel1* affect RA signaling, we administered *trans*-RA and its inhibitor (WIN18,466) in WT, *Pramex1* sKO, *Pramel1* sKO, and dKO mice at P2. We then evaluated the frequency of SCO tubules at P7 using whole-mount IFS with the TRA98 antibody (Fig. 6A). After RA treatment, the frequency of SCO tubules in WT, *Pramex1* sKO, *Pramel1* sKO, and dKO testis increased by 14%, 47%, 68%, and 73%, respectively (Fig. 6B), compared to the non-RA treatment group (Fig. 1B). This result indicated that RA treatment not only exacerbated the defect in the sKO and dKO mice but also affected the WT mice. Although the frequency of SCO tubules in either *Pramex1* sKO (11.43 ± 0.31%) or *Pramel1* sKO (12.95 ± 0.84%) was elevated after the RA treatment when compared to WT (1.91 ± 0.27%) (*P* < 0.01), it was significantly lower in the dKO mice (31.65 ± 0.82%) (*P* < 0.01) (Fig. 6A, B).

Upon inhibitor treatment, the frequency of SCO tubules in WT (1.51 ± 0.70%), *Pramex1* sKO (1.62 ± 0.08%), and *Pramel1* sKO mice (1.86 ± 0.86%) decreased to the baseline, suggesting that the SCO phenotype was recovered in the sKO mice. In contrast, SCO tubules were still present in the dKO mice, although in a much lower frequency (7.20 ± 0.43%) (Fig. 6A, B), implying a partial recovery in the dKO mice. The results indicated that the responsiveness of exogenous RA was altered in the sKO and dKO mice, and the dosage of RA inhibitor (WIN18,466) used in this study was adequate to offset the impact of single gene deletion, but not adequate to the double gene deletion. Thus, this data suggested a synergistic enhancement of RA signaling in the dKO mice, which required a higher dosage of inhibitor to counteract.

To further elucidate the role of PRAMEX1 within the RAR signaling pathway, we carried out a co-immunoprecipitation (co-IP) experiment to verify the interaction between PRAMEX1 and RARα. Relative to the expression in the WT testis input sample, the presence of PRAMEX1 in the RARα co-IP products exhibited a 1.3-fold increase, suggesting an enrichment of PRAMEX1 within the RARα protein complex (Fig. 6C, D). Moreover, the expression of RARα also displayed a 1.5-fold stronger band in the PRAMEX1 precipitation in WT testis compared to input sample (Fig. 6C, D), whereas the negative control (YBX2) was only detected in the input sample but not in either RAR or PRAMEX1 precipitations (Fig. 6C, D). These results suggested a direct interaction between PRAMEX1 and RARα, similar to the interaction of PRAMEL1 with RARα (as demonstrated in the co-IP data in [24]). In line with Fig. 2, in *Pramex1* sKO mice, no PRAMEX1 was detected in the testis or precipitation samples (Fig. 6C). Conversely, *Pramel1* sKO mice displayed a 3.32-fold increase of PRAMEX1 in the RARα co-IP products and 2.48-fold increase of RARα in PRAMEX1 precipitation relative to testis sample (Fig. 6C, E). These results suggest that in the absence of *Pramel1*, a compensatory mechanism partially restores the interaction with RARα by upregulating the *Pramex1* gene. Our findings regarding RA and RAR signaling imply that this restoration occurs as the upregulated gene assumes the role of the deleted gene in repressing RAR signaling.

### Compensatory gene effects were observed in *Pramex1* and *Pramel1* sKO females but absent in dKO females

**Fig. 7:**
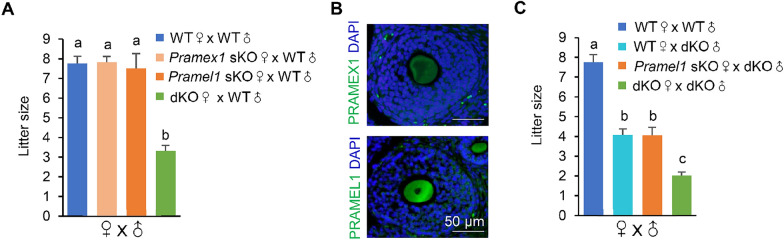
*Pramex1* and *Pramel1* exhibit compensatory effects on the fecundity of sKO female mice. **A** Litter size for female mating tests (n = 7 ~ 8). Data were expressed as mean ± SEM. Significance was assessed among the four mattings (WT♀ x WT♂, *Pramex1* sKO♀ x WT♂, *Pramel1* sKO♀ x WT♂, female dKO♀ x WT♂ mice). **B** IFS for PRAMEX1 and PRAMEL1 in ovary cross-section. Bar = 50 µm. **C** The litter size of different mattings suggests that the fecundity of both male and female *Pramex1/Pramel1* dKO mice was decreased (n = 7 ~ 8). Significance was assessed among the four mattings (WT♀ x WT♂, WT♀ x dKO♂, *Pramel1* sKO♀ x dKO♂, dKO♀ x dKO♂ mice) which producing 29, 30, 31, 40 litters from 7, 7, 8, 11 breeding pairs, respectively. Data were expressed as mean ± SEM. Values that do not share a common superscript (a–c) were found to differ significantly (*P* < 0.05)

The fecundity of *Pramex1* and *Pramel1* sKO female mice was examined by a female mating test, where mature *Pramex1* and *Pramel1* sKO female mice (2-months old, n = 7) were bred with mature WT male mice continually for 6 months. The WT female mice (2-months old, n = 7) served as controls. The litter sizes of the female *Pramex1* and *Pramel1* sKO mice (7.84 ± 0.28 and 7.52 ± 0.75) were not significantly different from the WT female (7.76 ± 0.38) (*P* = 0.43 and 0.33) (Fig. 7A), suggesting that the fecundity of both *Pramex1* and *Pramel1* sKO females was normal. Apparently, the ablation of either *Pramex1* or *Pramel1* did not have a visible effect on female fecundity. These results raised a question about whether the *Pramex1* and/or *Pramel1* are male-specific, as a previous report suggested both *Pramex1* and *Pramel1* predominantly expressed in the testis, but not in the ovary [14, 38]. We conducted IFS with the PRAMEX1- and PRAMEL1-specific antibodies on mature WT ovarian sections and observed a clear enrichment of both PRAMEX1 and PRAMEL1 proteins in the cytoplasm of oocytes in secondary and antral follicle (Fig. 7B). If PRAMEX1 and PRAMEL1 are expressed in oocytes, why did the *Pramex1* or *Pramel1* sKO females not show any reproductive phenotypes? One feasible explanation could be that the *Pramex1* and *Pramel1* genes possess compensatory functions with each other in female mice. To test the hypothesis, a mating test was performed for the *Pramex1/Pramel1* dKO females (bred with mature WT males for 6 months, n = 6). The average number of offspring in the dKO females was 3.31 ± 0.29, which was significantly lower than in *Pramex1* and *Pramel1* sKO (7.84 ± 0.28 and 7.52 ± 0.75) and WT females (7.76 ± 0.38) (*P* < 0.01) (Fig. 7A). The litter size of dKO females was 57% smaller than that of WT mice, paralleling the reduced fecundity observed in dKO male mice (Fig. 4D). These results indicated that the ablation of both *Pramex1* and *Pramel1* significantly affects the female reproductive capacity, confirming their compensatory role in female reproduction.

During the generation of dKO mice, a significantly lower-than-expected rate of dKO females was produced in the F2 (*P* = 4.52 × 10^–8^) and F3 generations (*P* = 1.44 × 10^–14^), respectively (Table 1). To explore the underlying causes, we conducted a thorough analysis of gamete genotypes produced by F1 and F2 animals and their male-to-female sex ratio among offspring. In F2, the sex ratio of the offspring was 1:1.04 as expected (Table 3). The F1 heterozygous males and females were expected to produce four different genotypes of sperm and eggs, yielding 16 different genotypes of offspring in F2 generation, with an anticipated distribution of 6.25% for each genotype under a normal breeding condition (Table 3). However, the observed distribution of genotypes differed significantly from the prediction (*P* = 4.52 × 10^–8^) (Table 1). Among the 296 pups analyzed, none of them were dKO females (*Pramex1*^*−/−*^*, Pramel1*^*−/−*^), while only 4 (1.35%) were dKO males (*Pramex1*^*−/Y*^*, Pramel1*^*−/−*^), which were significantly lower than the expected 6.25%. In contrast, 30 pups (10.14%) were WT males (*Pramex1*^+*/Y*^*, Pramel1*^+*/*+^), which were significantly higher than the expected rate (Table 3). The total number of pups produced from "*Pramex1*^*−*^*, Pramel1*^*−*^" eggs and sperm were 37 (12.5%) and 50 (16.9%), respectively, which were significantly lower than the expected 74 pups (25%) (*P* = 5.96 × 10^–6^) (Table 3). These results suggested that loss of homozygous dKO embryos occurred during the heterozygous male and heterozygous female dKO mating, and the Y-bearing "*Pramel1*^*−*^*, Y*" sperm had a less effect on embryonic survive than the X-bearing "*Pramex1*^*−*^*, Pramel1*^−^" sperm.

A total of 154 pups were produced in the F3 generation by mating heterozygous dKO females (*Pramex1*^*−/*+^*;Pramel1*^−/−^) with homozygous dKO males (*Pramex1*^*−/Y*^*;Pramel1*^−/−^), which produced two genotypes of eggs "*Pramex1*^*−*^*, Pramel1*^*−*^" and "*Pramex1*^+^*, Pramel1*^−^" and two genotypes of sperm, the X-bearing "*Pramex1*^*−*^*, Pramel1*^*−*^" and Y-bearing "*Pramel1*^*−*^*, Y*" sperm (Table 4). Consistent with the observation in the F2 generation, the number of homozygous dKO female and male pups produced from the "*Pramex1*^*−*^*, Pramel1*^*−*^" eggs was 9 (5.9%) and 10 (6.5%), respectively, significantly lower than the expected 25% (or 38.5 pups) (*P* = 1.44 × 10^–14^) (Table 4). In contrast, the single gene deletion "*Pramex1*^+^*, Pramel1*^*−*^" eggs produced 59 female (38.3%) and 76 male (49.4%) pups, significantly higher than the expected 25%. Furthermore, the male-to-female sex ratio was skewed (1:0.79) in this mating (*P* = 0.1469) (Table 4).

We further analyzed the litter size data from our mating records, including mature WT, *Pramel1* sKO, and dKO female mice bred with dKO males. As expected, the average litter size of WT♀ x dKO♂ (4.08 ± 0.30) was similar to *Pramel1* sKO♀ x dKO♂ (3.94 ± 0.42) (*P* > 0.05) (Fig. 7C). The dKO♀ x dKO♂ mating produced an average of 2.03 ± 0.18 pups/litter, which was approximately 50% smaller than the average litter size of sKO♀ x dKO♂ (*P* < 0.01) (Fig. 7C). It is worth noting that the dKO♀ x dKO♂ mating produced a total of 81 pups (42♂ and 39♀) with a male-to-female sex ratio of 1:0.93 (*P* > 0.05).

Taken together, our data suggested that the function of *Pramel1* was compensated by the *Pramex1* gene in the *Pramel1* sKO females. The function of "*Pramex1*^*−*^*, Pramel1*^*−*^" oocytes was affected more than X-bearing sperm "*Pramex1*^*−*^*, Pramel1*^*−*^". Therefore, embryos produced from this type of eggs had a less survival rate compared to those produced from "*Pramex1*^*−*^*, Pramel1*^*−*^" sperm. However, it was unclear if the lower-than-expected homozygous dKO pups found in this study was due to embryonic lethality (and loss) or germ cell loss during gametogenesis.

### Follicle quantity and early embryonic count decrease in *Pramex1/Pramel1* dKO females

**Fig. 8:**
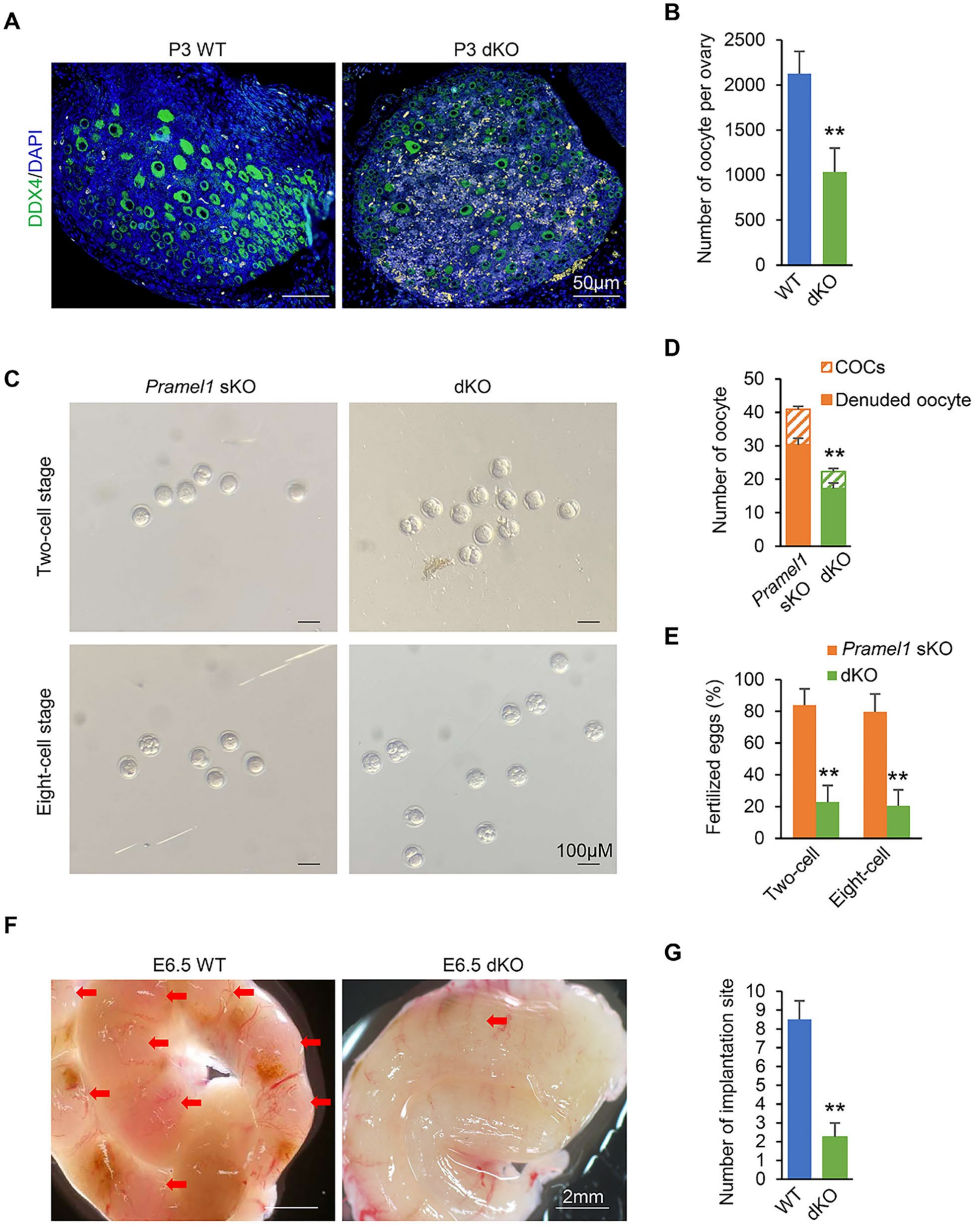
The *Pramex1/Pramel1* dKO females exhibit reduced number of follicles, which leads to embryo loss. **A** Representative images of IFS with DDX4 (green) in ovary cross-sections for WT and *Pramex1/Pramel1* dKO mice at P3 (n = 3). Bar = 50 µm.** B** Total number of follicles per ovary. The whole ovary was sectioned, and the counting was performed every five sections, and the number follicles was multiplied by five to determine the total number of follicles per ovary. **C**–**E** Results from in vitro fertilization (IVF) experiment. The quality of oocyte with genotyping of "*Pramex1*^+^, *Pramel1*^*−*^" and "*Pramex1*^−^, *Pramel1*^*−*^" were detected through in-vitro fertilize with "*Pramex1*^−^, *Pramel1*^*−*^" or "*Pramel1*^*−*^*,*Y" spermatozoa*.* **C** Representative figures of embryos (2-cell and 8-cell) after in vitro fertilization for *Pramel1* sKO and *Pramex1/Pramel1* dKO oocyte and dKO spermatozoa. Bar = 100 µm. **D** Number of denuded oocytes and cumulus-oocyte complexes (COCs) in each animal for *Pramel1* sKO and *Pramex1/Pramel1* dKO (n = 3). **E** Rate of 2-cell and 8-cell embryos from *Pramel1* sKO and *Pramex1/Pramel1* dKO oocytes. The rate was calculated by dividing the number of two-cells or eight-cells embryos by the number of COCs. **F** Representative images illustrating the uteri at E6.5 in WT and dKO mice, aimed at enumerating implantation sites. Bar = 2 mm. **G** The number of implantation sites in WT and dKO female at E6.5. Data were expressed as mean ± SEM. ** indicates statistically significant difference (*P* < 0.01)

To further explore the effects of the concurrent absence of PRAMEX1 and PRAMEL1 on oogenesis, we conducted a quantitative analysis of DDX4-positive primary follicles in dKO ovaries at P3. The *Pramex1/Pramel1* dKO females (1038.47 ± 261.91/ovary) exhibited a notable 51% reduction in the number of follicles compared to WT mice (2126.57 ± 249.75/ovary) (*P* < 0.01) (Fig. 8A, B). This reduction aligns with the 57% decrease in litter size observed in female dKO mice when compared to WT females. The results indicated that the double deletion of the two genes led to a decrease in the number of follicles per ovary.

To evaluate the effect of the two genotypes of eggs "*Pramex1*^−^, *Pramel1*^*−*^” and "*Pramex1*^+^, *Pramel1*^*−*^" on fertilization and early embryo development, we conducted in vitro fertilization (IVF) with oocytes collected from dKO and *Pramel1* sKO mice at P60. We assessed several parameters, including the number of cumulus enclosed and denuded oocytes, and the rate of embryos at two-cell and eight-cell stage. The total number of oocytes, including COCs and denuded oocytes, in the dKO group (22.33 ± 2.57/animal) was 45% lower than that in the sKO group (40.89 ± 2.43/animal) (*P* < 0.01). The proportion of oocytes contained in a COC was similar in both groups (dKO 23% and sKO 26%, *P* > 0.05) (Fig. 8C, D). This finding was consistent with our earlier results (Fig. 8A, B), demonstrating that dKO females exhibited a 51% decrease in follicle count within the ovaries. For IVF experiments, only oocytes within a COC were used. We found that the average number of dKO COC that were fertilized (2-cell) and progressed to the 8-cell stage were 1.22 ± 0.75 and 1.11 ± 0.70 while the number of 2-cell and 8-cell in sKO were 8.78 ± 0.87 and 8.33 ± 0.77 (Fig. 8C, E). In the dKO group, the rate (22.8 ± 10.54%) of 2-cells embryos was approximately 73% less than that in the sKO group (83.82 ± 10.35%) (*P* < 0.01) (Fig. 8E). Consistently, the rate of 8-cells embryos produced in the dKO group (20.58 ± 10%) was also significantly lower (74.2%) than that in the sKO mice (79.76 ± 11.18%) (*P* < 0.01) (Fig. 8E). In this study, we found that in vitro embryo productions rates from double-deletion male and female gametes were reduced at the two-cell stage, mirroring reduction in litter size observed in female dKO mating test.

In addition to evaluating early embryo development, we also examined the number of implantation sites in the uterus at E6.5 from a P60 female to determine the effect of double deletion. The uteri were obtained from WT and dKO females, while the sires of the embryos were WT and dKO males, respectively. The average number of implantation sites in the uteri of dKO female was 2.25 ± 0.74 which was significantly less (74%) than WT females (8.50 ± 1.00) (*P* < 0.01) (Fig. 8F–G). The number of implantation sites in the uteri of dKO females corresponds to the number of 2-cell and 8-cell embryos and the litter size in dKO mice (Fig. 8E and Fig. 7C).

In summary, the ovarian primary follicle count, in vitro embryo production rate, early embryo development and implantation analyses suggested that double deletion of *Pramex1* and *Pramel1* genes affects more on follicle quantity but less embryo development. In other words, loss of embryos during early embryo development in *Pramex1/Pramel1* dKO females was caused mainly by the reduction of primary follicles in the dKO ovary.

## Discussion

PRAME is known to regulate essential cellular processes, such as inhibition of differentiation and apoptosis, promotion of proliferation, and evading immune responses in cancer cells [25, 47, 48]. As one of the most amplified gene families, the multi-copy PRAME gene family exhibits widespread expression throughout both male and female reproductive life cycles, encompassing various developmental stages of gametogenesis and embryo development [10]. Recent studies on several members of the mouse Prame gene family, including *Pramel1, Pramel19, Pramex1* [16, 19, 23, 24], suggested that these proteins serve as inhibitors, repressing the RA/RAR signaling during germ cell development in spermatogenesis—a molecular mechanism previously reported for the role of PRAME in cancer cells [25, 47, 48]. Since the expression of these genes often overlaps in a particular stage of germ cells during gametogenesis, questions arise about the functional redundancy of these Prame members and whether they are complementary or interact with each other during gametogenesis. In this study, we addressed this question by examining two members, *Pramex1* and *Pramel1,* from the mouse Prame family. Previous work indicates that both *Pramex1* and *Pramel1* are predominantly expressed in the testis, particularly in spermatogenetic cells and mature spermatozoa [14, 38]. At the subcellular level, PRAMEX1 and PRAMEL1 are detected primarily in the nucleus and cytoplasm, including rER, Golgi apparatus, germ granular (particularly in chromatoid body), acrosome and sperm tail [38]. At the molecular level, both *Pramex1* and *Pramel1* are involved in the RA/RAR signaling to regulate spermatogonia proliferation, differentiation, and apoptosis during the establishment of the first round, and subsequent rounds of spermatogenesis in mice [23, 24]. However, their genetic interactions remain unknown. In the present study, we investigated their genetic interaction by charactering a *Pramex1/Pramel1* dKO mice model in comparison to the corresponding single gene KO and WT mice.

We found that the phenotypes, including testis weight, sperm count, rate of SCO tubules and litter size, in the *Pramex1/Pramel1* dKO male mice were lower than the *Pramex1* and *Pramel1* sKO and WT mice, but also significantly below the referenced values based on an additive genetic model (Figs. 4, 5), which assumes no genetic interaction [39–41]. The pronounced defects observed in *Pramex1/Pramel1* dKO males suggested that the interaction between *Pramex1* and *Pramel1* follows the genetic synergistic enhancement [44–46]. Synergy arises when the combined impact of two mutations on the phenotype of a double mutant exceeds reference derived from the additive effects of individual mutations [49]. Such interactions often result from mutations in functionally related genes, as seen in Toll-like receptor (TLR)-induced responses to multiple ligands (TLR1/2, 2/6, 4, 5, 7/8) and diverse cytokine secretion (IL-1α, IL-1β, IL-4, IL-6, IL-10) [49, 50]. Previous research in mice has reported synergistic enhancement of germ cell-expressed genes on male fertility [51]. Kierszenbaum et al. [51] found that spermatogenesis and fertility remained unaffected when male mice had one of these four genes knocked out: *Tnp2* (transition protein 2), *Acr* (acrosin), *H1.1* (histone H1.1), *H1t* (histone H1t) and *Smcp* (sperm mitochondria-associated cysteine-rich protein) [52–56]. However, *Hist1h1a/Mcsp, Hist1h1t/Mcsp, Tnp2/Mcsp* and *Acr/Mcsp* dKO male mice exhibited subfertility or near infertility [51]. These findings align with the data obtained from *Pramex1* and *Pramel1* sKO and dKO mice in this work, including concerning oogenesis and female fertility. No defects in oogenesis and fertility were observed in *Pramex1* or *Pramel1* sKO female mice. However, a significant reduction in the number of developing follicles, preimplanted embryos, and litter size was noted in *Pramex1*/*Pramel1* dKO females. These results indicated that the loss-of-function of *Pramex1* (or *Pramel1)* in the single gene mutant females is entirely compensated by the presence of the other gene, *i.e. Pramel1* (or *Pramex1*). The pronounced defects observed in the dKO females indicated synergistic effects of the two genes during oogenesis and female fertility [49]. In contrast to the complete functional compensation of the *Pramex1* and *Pramel1* genes during oogenesis in the sKO females, partial compensation was evidenced in the sKO males by the upregulation of the protein expression in the other non-mutated gene and the distinct phenotypes associated with *Pramex1* or *Pramel1* sKO mutants during spermatogenesis [23, 24]. The differences in *Pramex1* and *Pramel1* compensation between female and male sKO mice could be attributed to mechanism of how RA is involved in the processes of spermatogenesis and oogenesis. RA plays a primary role in initiating meiosis during oogenesis, while RA controls three essential transitions of germ cells from undifferentiated to differentiated spermatogonia, meiosis and spermatid elongation during spermatogenesis [26–28, 34, 57]. Data from previous reports [10, 23, 24] and this work indicated that *Pramex1* and *Pramel1* genes function on different development stages of spermatogenic cells through RA signaling, implying that these two genes are not redundant during spermatogenesis. However, their functions may influence the same stage of germ cells during oogenesis, as RA signaling primarily functions to initiate meiosis in ovary. Hence, the ablation of either one of the two genes in female could be fully compensated by the other gene, resulting in no observable defects in sKO females.

In mattings involving heterozygous and homozygous *Pramex1/Pramel1* dKO mice, "*Pramex1*^−^, *Pramel1*^*−*^” eggs produced significantly fewer homozygous dKO female pups, showing a skewed male-to-female sex ratio. On the other hand, "*Pramex1*^+^, *Pramel1*^*−*^” and "*Pramex1*^−^, *Pramel1*^+^” eggs yielded more heterozygous dKO pups than expected. Although "*Pramex1*^+^, *Pramel1*^*−*^” and "*Pramex1*^−^, *Pramel1*^+^” eggs, as the single gene mutants, produced a comparable number of pups to wild-type "*Pramex1*^+^, *Pramel1*^+^” eggs due to gene compensation (see discussion above), the observed loss of dKO female embryos with a homozygous "*Pramex1*^−/−^, *Pramel1*^*−/−*^” genotype could suggest embryo lethality. However, our observation challenges the embryo lethality hypothesis, as "*Pramex1*^−^, *Pramel1*^*−*^” eggs and "*Pramex1*^−^, *Pramel1*^*−*^” sperm produced live female dKO mice. Therefore, understanding the mechanism governing the functional interaction between *Pramex1* and *Pramel1* during oogenesis and fertilization warrants further investigation.

It was previously suggested that various members of the Prame family may play distinct roles at different stages of germ cell development. While each member may have a minor or non-essential function in spermatogenesis, the entire family collaborates to regulate the three major transitions of spermatogenic cells through the repression of RA/RAR signaling [24]. However, it is unknown if there is a dosage effect on the inhibition of the RA signaling by different Prame members. Upon the exogenous RA treatment, the frequency of SCO tubules was increased in both sKO and dKO testes, as well as the WT testis, suggesting that the presence and the severity of SCO phenotype are sensitive to the RA level. The dosage of RA inhibitor used in the present study was sufficient to counteract the effect of loss-of-function of RA repressor, PRAMEX1 (or PRAMEL1), in the sKO mice, but not sufficient to offset the effect of loss of both repressors, PRAMEX1 and PRAMEL1, in the dKO mice. This data suggested that a synergistic enhancement of RA signaling occurs in the dKO mice, and different members of the Prame family may function in a dosage manner to fine-tunes RA signaling [24].

## Conclusions

This study revealed the double deletion of *Pramex1* and *Pramel1* comparably decreased the fecundity of both male and female mice by approximately 50%, which was severe than phenotypes observed in *Pramex1* or *Pramel1* single gene mutants. It indicated a synergistic interaction between the two members of the Prame family during gametogenesis. The *Pramex1* and *Pramel1* fully compensate for each other during oogenesis, but only partially compensate during spermatogenesis. This difference in gene compensation is likely due to the dosage effect of *Pramex1* and *Pramel1* in repressing RA signaling, and the variation in RA function between oogenesis and spermatogenesis. These findings contributed to new knowledge about the Prame family role in reproduction and encouraged the study of the other members and their interactions in the Prame family, shedding light on our understanding of the multi-copy gene families in germ cell formation and function.

## Data Availability

All data generated or analyzed during this study are included in this published article.

## Abbreviations

Acr: Acrosin
Actb: Actin, beta
BSA: Bovine Serum Albumin
CB: Chromatoid body
Cdkn1a: Cyclin-dependent kinase inhibitor 1a
CNVs: Copy number variations
CO^2^: Carbon dioxide
COC: Cumulus-oocyte complex
Co-IP: Co-immunoprecipitation
CTAs: Cancer/testis antigens
CYP26B1: Cytochrome P450 family 26 subfamily
DAPI: 4',6-Diamidino-2-Phenylindole
DAZ: Deleted in AZoospermia
DDX4: DEAD-Box Helicase 4
dKO: Double knockout
DMRT: Double-sex and mab-3 related transcription factor
ESCs: Embryonic stem cells
E + number: Embryonic day + number
H1.1: Histone H1.1
H1t: Histone H1t
HTF: Human tubal fluid
ID4: Inhibitor Of DNA Binding 4
IFS: Immonofluorescent staining
IL: Interleukin
IMC: Intermitochondrial cement
IVF: In vitro fertilization
KO: Knockout
KSOM: Potassium Simplex Optimization Medium
LRR: Leucine rich repeat
MEM: Minimum Essential Medium
MZF1: Myeloid zinc finger1
N^2^: Nitrogen
NC: Negative control
O^2^: Oxygen
P + number: Postnatal day + number
PBS: Phosphate-buffered saline
PCR: Polymerase chain reaction
PRAME: Preferentially expressed antigen in melanoma
PFA: Paraformaldehyde
PMSG: Pregnant Mare Serum Gonadotrophin
Pramel1: Prame like 1
Pramex1: Prame like, X-liked 1
PRC2: Polycomb repressive complex 2
qRT-PCR: Quantitative real-time PCR
RA: Retinoic acid
RA/RAR: Retinoic acid/retinoic acid receptor
RARα: Ratinoic acid receptor alpha
Rpl22: Ribosomal protein L22
Rpl22l1: Ribosomal protein L22-like 1
RRM: RNA-Recognition Motif
RT: Reverse transcription
SCO: Sertoli cell only
SEM: Standard error of mean
sKO: Single knockout
Smcp: Sperm mitochondria-associated cysteine-rich protein
SOX9: SRY-box transcription factor 9
Sry: Sex-determining region Y
TBS: Tris-buffered saline
TBS-T: Tris-buffered saline supplemented with 0.1% Tween 20
TDRD: Tudor domain-containing proteins
TLR: Toll-like receptor
Tnp2: Transition protein 2
TUNEL: Terminal deoxynucleotidyl transferase dUTP nick end labeling
WB: Western Blot
WT: Wild-type
YBX2: Y-Box Binding Protein 2
